# Chinese medicine boosts regenerative medicine in stem cell - based therapy

**DOI:** 10.1186/s13287-025-04618-6

**Published:** 2025-09-25

**Authors:** Zijuan Bi, Hongming Tang, Enkang Wang, Yinshu Wang, Yangyang Meng, Jianye Yuan, Zhongmin Liu

**Affiliations:** 1 Translational Medical Center for Stem Cell Therapy & Institute for Regenerative Medicine, Shanghai East Hospital, Tongji University School of Medicine, Shanghai, China; 2 Department of General Medicine, Beicai Community Health Service Center of Pudong New Ares, Shanghai, China; 3 Department of Traditional Chinese Medicine Surgery, Changhai Hospital), School of Traditional Chinese Medicine, The First Affiliated Hospital of the Navy Medical University, Naval Medical University, Shanghai, China; 4 Department of Spleen and Stomach Diseases, Yangpu District Traditional Chinese Medicine Hospital in Shanghai, Shanghai, China; 5 Longhua Hospital, Shanghai University of Traditional Chinese Medicine, Shanghai, China

**Keywords:** Regenerative medicine, Stem cell, Chinese medicine (CM), Mechanism, Senescence, Environment, Proliferation and differentiation, Survival, Exosome

## Abstract

This review article explores the possible role of Chinese medicine (CM) in modulating stem cells for regenerative medicine, synthesizing evidence from animal experiments and human trials. The article focuses on how CM modulates the stem cell environment, specifically their roles in delaying cellular senescence and promoting stem cell survival, enhancing proliferation and differentiation, as well as stimulating exosome secretion. It also conducts a critical analysis of methodological rigor and clinical transparency within the included studies to enable a more objective assessment of their reliability and reproducibility. To guarantee the responsible integration of CM and stem cells in future clinical application, it also discussed the safety, efficacy, and heterogeneity of stem cells, as well as delivery methods, alongside the dose-response relationship of CM. The current evidence for CM in stem cell therapy remains constrained by the absence of standardized comparative baselines in animal studies and clinical outcome assessment. This methodological gap not only compromises the evidentiary weight of herbal effects but also introduces confounding variables in studies. The elucidation of CM mechanistic role in stem cell therapeutics necessitates robust interdisciplinary collaboration, this is an imperative and critically urgent thing within peer-reviewed research framework.

## Introduction

Regenerative medicine is a biomedical discipline aimed at repair or replacement of damaged, diseased, or metabolically deficient organs, tissues, and cells via tissue engineering, cell transplantation, artificial organs or bioartificial organs and tissues [1]. Stem cell-based therapy is a branch of regenerative medicine, wherein stem cells are able to differentiate into various type of cells under specific conditions. Stem cells have shown broad prospects in the field of regenerative medicine. Through asymmetric division, they not only replenish the stem cell pool but also differentiate into tissue-specific cells to perform particular physiological function in the growth, development, repair, and regeneration of the organism. Their effectiveness in tissue repair and regeneration is remarkable, making them a hotspot in medical research. Crucially, distinctly stem cell lineages exhibit divergent biological properties that directly impact their clinical translation. Embryonic stem cells (ESCs) have always been controversial due to ethical concerns regarding their source, which also limits their application in clinical research. Moreover, preclinical studies have found that ESCs lack immune privilege and are subject to immunological rejection [2]. Additionally, the risk of malignancy further restricts ESCs-based treatment [3]. Induced pluripotent stem cells (iPSCs), an alternative to ESCs, can be reprogrammed from adult somatic cells (e.g., fibroblasts, epidermal cells) and differentiate into neural cells [4] and cardiomyocytes [5], thereby ushering in an era of personalized medicine in modern medicine. Mesenchymal stem cells (MSCs), characterized by their abundant sources [6], low immunogenicity, and immunomodulatory properties [7], have become a vital component in regenerative medicine. Additionally, other types of adult stem cells, such as intestinal stem cells (ISCs) [8], hair follicle stem cells (HFSCs) [9], and neural stem cells (NSCs) [10], are responsible for the growth, repair, and regeneration of specific tissues. These stem cells have the potential to release various bioactive factors, regulate the tissue microenvironment, and promote the repair of damaged tissues. However, studies often exhibit low retention rate and immune rejection across the program, and safety issues need to be taken seriously before they can be used in clinic [11]. Given the obstacles in stem cell therapy, Chinese medicine (CM) could provide multi-target interventions to attenuate these effects. Existing studies have found that CM may support stem cell-based therapy by enhancing the survival rate, promoting migration and retention, stimulating angiogenesis, reducing immune rejection, guiding directional differentiation, and remarkably improving the proliferation and differentiation capabilities of stem cells.

CM comprises a sophisticated amalgamation of multiple bioactive chemical constituents, inherently accounting for its multifaceted mechanisms of action. Under the theory guidance of traditional Chinese medicine (TCM), CM can be used to prevent and treat diseases, as well as regulate the overall balance of yin and yang (阴阳平衡). According to modern science research, the active components of CM encompass alkaloids (e.g., berberine, rhynchophylline, and ephedrine), volatile oils (e.g., menthol, eugenol, and camphor), and glycosides (e.g., ginsenosides, notoginsenosides, and baicalin). These bioactive compounds modulate stem cell behaviors, like proliferation, differentiation, anti-inflammation, and anti-oxidation. The continued use of these medicines is attributed to their remarkable clinical efficacy. Current studies demonstrate that CM plays a significant role in regenerative medicine through the regulation of specific signaling pathways [12], maintenance of systemic homeostasis [13], and improvement of stem cell engraftment efficiency [14]. Preclinical studies have provided preliminary evidence suggesting synergistic effects when combining CM with MSCs. Ma et al. [15] observed enhanced bone marrow mesenchymal stem cell (BMSC) colonization in liver tissue, reduced hepatocyte apoptosis, suppressed pathological injury, and decreased fibrosis markers when combining BMSCs with Bushen Huoxue Huazhuo Formula (补肾活血化浊方) in a liver fibrosis model, surpassing monotherapy effects. This could plausibly involve the formula’s components improving the fibrotic hepatic niche, potentially by reducing oxidative stress [16], inhibiting key pro-fibrotic pathways [17] (e.g., TGF-β1), or promoting pro-regenerative chemokine expression [18], thus facilitating BMSC engraftment and paracrine activity. This illustrates a recurring theme in the CM-stem cell combination literature, intriguing synergistic efficacy in animal models contrasted by a significant lack of mechanistic depth. While correlating combination therapy with improved outcomes, the studies primarily demonstrate association rather than establishing detailed molecular causation or defining the specific interactive pathways between CM components and stem cell. While compelling preclinical data suggests enhanced therapeutic potential, the conspicuous absence of well-designed clinical trials validating efficacy, safety, and mechanistic hypotheses in human subjects renders the direct clinical significance of such combination uncertain. The above promising synergy remains confined to proof-to-concept animal studies, necessitating rigorous translational research to bridge this critical gap before clinical application can be considered.

Beyond the potential synergistic effects observed with administering CM alongside exogenous MSCs, a growing body of research explores how CM components themselves, through their characteristic multi-targeting actions, can directly modulate endogenous stem cell population and influence their fate across diverse tissue. For instance, Liangxue Guyuan Yishen decoction (凉血固元益肾汤), was shown to induce ISC proliferation in a radiation-induced intestinal injury mouse model, potentially via increasing *Akkermansia muciniphila* abundance and facilitating short-chain fatty acids (SCFAs) secretion [19], although the precise SCFA mechanisms remain unclear. CM with blood-activating and stasis-removing (活血化瘀) effects demonstrates the potential to improve stem cell survival, inhibit apoptosis, and promote proliferation, differentiation, and migration in myocardial infraction contexts [20]. Shi-Bi-Man (石碧曼) promoted lactate dehydrogenase A (LDHA) metabolism in HFSCs in a primate model [21], while Bazi Bushen (八子补肾) was found in mice to regulate the balance of apoptosis and autophagy in epidermal stem cells (EpiSCs), mitigate aging signs [22]. Individual CM-derived compounds also show significant effects. Genipin (from the *Gardenia jasminoides*), accelerated nerve regeneration [23]. Cryptotanshinone (from *Salvia miltiorrhiza* ) profoundly impacted various stem cell populations, potentially improving therapy for cognitive disorders, Parkinson’s disease (PD), spinal cord injuries (SCI), obesity, and tumors [24]. Catalpol (prominent in *raw rehmannia*) enhanced MSC migration and recruitment for cartilage repair [25]. Baicalin (from *Scutellaria baicalensis*) influenced stem cell metabolism and differentiation fate relevant to menopausal syndrome therapy [26]. Although demonstrating the promoting effects of CM in stem cell-based therapy, some of these studies similarly lack exploration of deeper mechanisms, limiting evidence-based scientific explanations. Furthermore, the validity of animal data for human application remains a significant challenge.

**Fig. 1:**
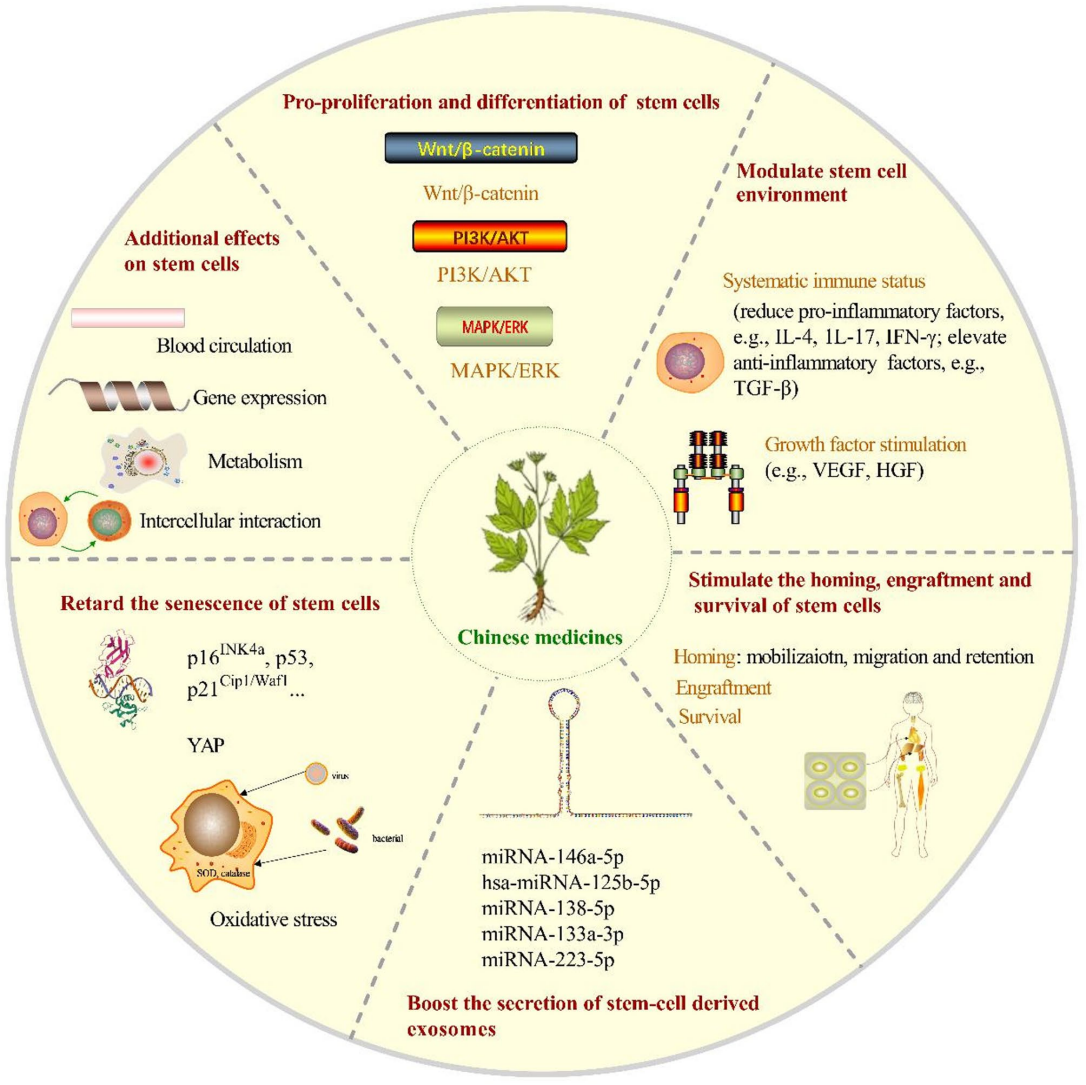
The multi-effects of CM on stem cells. Proliferation and differenation: CM promotes stem cell proliferation and differenation through wnt/β-catenin, PI3K/AKT, MAPK/ERK pathways; Stem cell environment: CM contributes to the improvement of stem cell environment by modulating systematic immune status and growth factor; Homing, engraftment and surival: CM improves the homing, engraftment and survival of stem cells; Stem-cell derived exosomes: CM influences the secretion of stem cell-derived exosomes to modulate intercelluler communication; Senescence of stem cells: CM delays the senescence of stem cells under oxidative stress, and supress aging-related peroteins expression; Additional effects: additional effects of CM on stem cells include gene expression, metabolism and cell interaction

Therefore, we searched Pubmed recently five years and CNKI using the following search term: (“stem cells” OR “stem cell” OR “progenitor cell” OR “progenitor cells”) AND (“tradtional Chinese medicine” OR “Chinese medicine” OR “Chinese medicines” OR “herb” OR “herbs”). This review attempts to present the role of CM in regulating the environment of stem cells [27], supressing inflammation [28], and tissue repair [29] from the latest research, and to analyze the underlying mechanisms behind these effects as much as possible (Fig. 1), as well as the problems existing in the research. It also presents a brief table of the research content for each part to facilitate clear understanding for readers. It aims to provide valuable insights for further research on the combined application of CM and stem cells in regenerative medicine and to promote interdisciplinary collaboration in this field to achieve better therapeutic outcomes and health management.

## CM modulates stem cell environment

The stem cell environment encompasses the specific microenvironment (niche) and broader systemic conditions that influence cell behavior. The niche is a dynamic and complex system maintaining stem cells through interactions involving cellular and non-celluler components, supporting self-renewal while mudulating maturation and functional potential [30, 31]. CM formulations demonstrate potential for modulating this environment. Clinical evidence suggested kidney-tonifying (补肾) strategies promoted liver regeneration and repair in chronic hepatitis B-related liver failure, potentially affecting stem cells and their microenvironment [14]. However, the research lacked a double-blind design and may be susceptible to observer bias. Similarly, the herbal compound Diwu Yanggan (地五养肝) modulated liver regeneration by restoring IL-1, growth-regulated oncogene/keratinocyte chemoattractant (GRO/KC), and vascular endothelial growth factor (VEGF) levels, thereby influencing the hepatic stem cell microenvironment in an experimental rat model of liver injury [32]. Such modulation fosters a more favorable microenvironment supporting hepatic tissue structure and function.

Besides, CM exhibits systemic immunomodulatory properties [33, 34], which can enhance stem cell surival [35] and improve the therapeutic environment. In an allergic rhinitis (AR) animal model, qi-fang-bi-min-tang (芪防鼻敏汤)-treated MSCs significantly reduced pro-inflammatory factors (e.g., IL-4, IL-17, IFN-γ, histamine, and IgE), increased splenic Treg cell proportions, and elevated plasma TGF-β1 levels compared to untreated MSCs [36]. Similarly, in an ischemic model, combined curcumin and human umbilical cord mesenchymal stem cell (hUC-MSC) therapy outperformed either monotherapy, significantly supressing pro-inflammatory cytokins (e.g., IL-1β, TNF-α, IL-6) and oxidative mediator (e.g., MDA), while boosting anti-inflammatory cytokins (e.g., TGF-β1, IL-10) and antioxidant activity (e.g., SOD and GPx) [37]. While these studies demonstrate CM’s impact, notable limitations include that failure to isolate CM effects from stem cell activity, lack of detailed phytochemical characterization (e.g., HPLC fingerprint) for quality control, and absence of long-term efficacy data. These gaps require resolution to confirm causality and clinical relevance.

Growth factors critically shape the niche by binding cell surface receptors to regulate proliferation, differentiation, and survival. Their increased expression can elevate stem cell density and functional efficacy [38]. CM enhances several key growth factor, like hepatocyte growth factor (HGF), VEGF, fibroblast growth factor (FGF), insulin-like growth factor 1 (IGF-1), platelet-derived growth factor (PDGF), and bone morphogenetic protein 2 (BMP-2). Clinical research indicated combing Lugua polypeptides (鹿瓜多肽) with UC-MSCs improved rheumatoid arthritis (RA) outcomes by significantly increasing HGF expression [39]. However, the control group in the research only used methotrexate and leflunomide, without comparison to other CM formulations, impacting scientific validity. VEGF promotes endothelial cell proliferation and migration, altering the niche vasculature and potentially affecting stem cell function and fate. CM promotes VEGF expression [40]. Combining Naomai Yihao (脑脉益好) capsules with VEGF gene-modified BMSCs significantly enhanced angiogenesis, eatablishing an vital blood supply to sustain transplanted stem cells [41]. Post-myocardial infarction (MI), ischemia/hypoxia and fibrosis creats a hostile microenvironment. This triggers FGF signaling (via FGFR2 binding) exerting anti-fibrotic effects, and induces hypoxia-inducible fator 1α (HIF-1α), stimulating VEGF secretion to promote capillary formation. Luo et al. [42] demonstrated that combing iPSC-derived with modified Taohong Siwu decoction (桃红四物汤) significantly improved cardiac function parameters cardiomyocyte (e.g., EF, FS, LVIDs, LVIDd, LVEDV, LVESV) and reduced infarct size, collagen content, and wall thickness in an MI mouse model. These improvements correlated with increased VEGF and FGF levels, likely attributable to CM’s blood-activating and stasis-resolving (活血化瘀) properties improving hemodynamics and optimizing cell retention. IGF-1 likewisely enhances neovascularization. In a late-stage hypertension rat model, combined adipose-derived mesenchymal stem cells (ADSCs) and Danggui (当归) augmented cardiac funcion, potentially via IGF-1 upregulation [43]. But the active constituents within Danggui extract responsible for IGF-1 modulation were not identified, nor did it control for potential confounding contributions from sponteneous IGF-1 secretion by ADSCs. PDGF receptors are crucial for neovascularization and MSC recruitment/differentiation [44]. Shengmaiyizhi decoction (生脉益智汤) ameliorated memeory and cognitive impariment in a multi-infarct dementia model, linked to increased PDGF-β receptor mRNA levels [45]. However, the study failed to address critical mechanistic questions, including whether downstream protein expression was concurrently upregulated and whether receptor phosphorylation-mediated activation occurred. And icariine enhanced BMP-2 production [46], a key osteoinductive factoe for bone regeneration [47].

CM optimizes the stem cell niche primarily by promoting immunomodulation [48] and the secretion of key growth factors via multiple pathways. This establishes a more stable, supportive environment enhancing stem cell survival, function (including improved immunoregulation and longevity), and ultimately, therapeutic efficacy. The synergy betweem CM and stem cells represents a promosing comprenhensive approach. However, the precise mechanistic interplay require further elucidation. Key unresolved questions include whether CM components directly modulate growth factor secretion to influence stem cell function or act on stem cells to induce autocrine/paracrine factor production. Compelling evidence suggests both pathways may contribute bidirectionally. More rigorous experimental designs are required to demonstrate CM-induced growth factor secretion as the primary mechanism for its beneficial effects on stem cells. Researches on CM modulation of the stem cell environment is summarized in Table 1.

**Table 1:** CM modulation on stem cell environment

Cell type	CM	Diseases/Models	Effects	References
Hepatic stem cell	Diwu Yanggan (地五养肝)	Liver injury-regeneration impairment (partial hematectomy rat models)	Modulating the hepatic microenvironment (increased ratio of CD34/CD45 double-positive cells and restoration of IL-1, GRO/KC, and VEGF levels to normal)	[32]
MSCs	qi-fang-bi-min-tang (芪防鼻敏汤)	AR animal models	Decreased levels of IL-4, IL-17, IFN-γ, histamine, and IgE, along with increased Treg cells proportions, and TGF-β1 levels in plasma	[36]
hUC-MSCs	Curcumin	Ischemic animal models	Decreased pro-inflammatory cytokines (IL-1β, TNF-α, IL-6) and lipid peroxidation marker (MDA), with increased anti-inflammatory cytokines (IL-4, TGF-β1, IL-10) and antioxidation enzymes (SOD, GPx)	[37]
UC-MSCs	Lugua polypeptides (鹿瓜多肽)	RA patients	Promoting HGF secretion	[39]
BMSCs	Naomai Yihao (脑脉益好)	Cerebral ischemic tissues in rat models	Promoting angiogenesis	[41]
iPSC-derived cardiomyocyte	modified Taohong Siwu decoction (桃红四物汤)	MI mouse models	Increased levels of VEGF and FGF, enhanced systolic function (EF, FS) with reverse remodeling (LVIDs, LVEDV) and attenuated pathology (infarct size, collagen content, wall thickness)	[42]
ADSCs	Danggui (当归)	Late-stage hypertension rat model	Upregulation of IGF-1 expression	[43]
hBMSCs	Icariine	hBMSCs are induced directionally to obtain human osteoblasts	Bone regeneration (enhanced BMP-2 production)	[46]

Abbreviarions: CM, Chinese medicine; AR, allergic rhinitis; MSCs, mesenchymal stem cells; RA, rheumatoid arthritis; hUC-MSCs, human umbilical cord mesenchymal stem cells; BMSCs, bone marrow mesenchymal stem cells; iPSC, pluripotent stem cells; MI, myocardial infarction; ADSCs, adipose-derived mesenchymal stem cells; HGF, hepatocyte growth factor; VEGF, vascular endothelial growth factor; FGF, fibroblast growth factor; IGF-1, insulin-like growth factor 1; BMP-2, bone morphogenetic protein 2

## CM delays stem cell senescence

Aging reprsents a complex biological process characterized by progressive decline in physiological function and reduced resilience to stressors. Key pathological features include stem cell exhaustion [49], diminished cellular proliferation [50], metabolic dysfunction [51], and imparied tissue repair [52]. At the molecular level, stem cell senescense involves epigenetic modefications, proteostatic dysfunction, and the detrimental influence of systemic factors like chronic inflammation, metabolic disregulation, and circadian rhythm disturbances [53]. Emerging evidence highlights the potential of CM to delay aging and aging-associated pathologies (e.g., cardiovascular diseases, diabetes, and cancer) partly by mitigating stem cell senescence and enhancing stem cell viability [54].

CM formulations counteract senescence by modulating critical signaling pathways. CM inhibits key senescence drivers like p^16INK4a^, p53, and p21^Cip1/Waf1^. Guilu Erxian Jiao (龟鹿二仙胶) significantly reduced p^16INK4a^, p53, and p21^Cip1/Waf1^ proteins expression, while increasing CDK2, CDK4, and hypophosphorylated pRb levels in chemotherapy-induced senescent hematopoietic stem cells (HSCs), thereby ameliorating their aging phenotype [55]. This effectively counters the sustained Rb hypophosphorylation that promotes cell cycle arrest. Liuwei Dihuang pills (六味地黄丸) attenuated ovariectomy-induced bone loss by alleviating BMSC senescence via activation of Yes-asssociated protein (YAP)-autophagy axis [56]. Activated YAP enhances autophagic clearance of senescent factors and suppresses the p53/p21 pathway. Downregulation of the transcription factor Nrf2 impairs antioxidant defenses (e.g., HO-1, NQO1, SOD, GPX), resulting in reactive oxygen species (ROS) accumulation, macromolecular damage, and senescence pathway activation. Zuogui Pills (左归丸) targeted Nrf2 activation in ovarian aging models, enhancing antioxidant enzyme activity and reducing ROS accumulation in ovarine germline stem cells (OGSCs) [12]. Erxian decoction (二仙汤) induced plasma exosome secretion in rats, these exosomes rescued impaired mitophagy in senescent BMSCs in vitro without compromising osteogenic potential [57]. However, the specific cellular sources of CM-induced exosomes remain unidentified. Ultraviolet radiation generates mitochondrial ROS and DNA damage, activating p38 MAPK and p53 pathways. Phosphoryated p53 (p-p53) and p-p38 induce p21-mediated cell cycle arrest and upregulate metalloproteinases (MMPs), degrading skin matrix components. Si Jun Zi decoction (四君子汤) inhibited p53, p-p53, and p21 expression, decreased p38 phosphorylation, and upregulated stem cell markers in aging EpiSCs [58]. Bioactive CM components (e.g., glycyrrhizic acid, ginsenosides Rg5/Rh2, liquirtin, pachymic acid C, atractylenolide II) effectively suppress ROS and modulate senescence markers in aging skin models. Ginseng-Sanqi-Chuanxiong (人参-三七-川芎) extracts reduced p53, p21, and p16 expression in senescent endothelial progenitor cells (EPCs) from D-galactose (D-gal)-induced vascular models, while enhancing EPC proliferation, migration, adhesion, secretory functions, and endothelial health [59]. Telomere damage initiates Klotho promoter methylation, downregulation this anti-aging protein. Klotho cooperates with telomerase reverse transcriptase (TERT) to suppress mitochodrial ROS and stabilize telomeres. Artemisia argyi water extract upregulated Klotho/TERT in doxorubicin-treated hADSCs, elongating telomeres and reducing mitochondrial superoxide [60].

In addition to the above-mentioned methods to influence cellular aging, CM can also protect against organelle dysfunction driving senescence. Mitochondrial dysfunction creats ROS, damaging mitochondria further in a vicious cycle. The mitochondrial proteostasis system, involving factors like Tid1 (Hsp40/DNAJA3) and receptor Tom20, is crucial for clearing misfolded proteins. Ohwia caudata extract upregulated Tid1 and Tom20 in doxorubicin-treated MSCs, restoring mitochondrial function, reducing ROS, stabilizing membrane potential, and decreasing apoptosis [61]. Endoplasmic reticulum (ER) stress, triggered by unfolded protein accumulation, activates the unfolded protein response (UPR). Persistent UPR induces apoptosis. Apoptotic cells release damage-associated molecular patterns (DAMPs), damaging the ISC niche and accelerating aging. Total ginsenosid inhibited the IRE1-JNK pro-apoptotic UPR pathway in *Drosophila* ISCs, preserving stem cell function [62].

Stem cell exhaustion is a central pillar of organismal aging, driving functional decline at tissue, organ, and systemic levels [63]. CM offers a promising multi-target strategy to combat stem cell seneacence. By modulating key pathway, enhancing antioxidant defenses, protecting organelle function, and acting through specific mechanisms relevant to different stem cell types, CM helps maintain stem cell activity, function, and regenerative capacity, thereby slowing tissue aging. Preclinical and clinical evidence support CM’s anti-aging effects via antioxidative, anti-inflammatory, organ-protective, and mitochondrial-stabilizing properties [64]. Further research in needed to fully elucidate these complex interactions. Table 2 summarizes key findings on CM delaying stem cell senescence.

**Table 2:** CM anti-senescence effects on stem cells

Cell type	CM	Diseases/Models	Effects	References
HSCs	Guilu Erxian Jiao (龟鹿二仙胶)	Chemotherapy-induced models	Downregulation of p^16INK4a^, p53, and p21^Cip1/Waf1^, upregulation of CDK2, CDK4, and pRb	[55]
BMSCs	Liuwei Dihuang pills (六味地黄丸)	Ovariectomy-induced bone loss models	Activation of Yes-sssociated protein (YAP)-autophagy axis	[56]
OGSCs	Zuogui Pills (左归丸)	Cyclophosphamide-induced ovarian aging models	Nrf2 activation, upregulation of antioxidant enzyme activity, reduction of ROS	[12]
BMSCs	Erxian decoction (二仙汤)	H_2_O_2−_induced aging models	Exosomes secretion induction, mitophagy restoration	[57]
EpiSCs	Si Jun Zi decoction (四君子汤)	Ultraviolet radiation-induced models	Inhibition of p53, p-p53, and p21 expression, decreased p38 phosphorylation, upregulated stem cell markers	[58]
EPCs	Ginseng-Sanqi-Chuanxiong (人参-三七-川芎) extracts	D-galactose (D-gal)-induced vascular models	Reduction of p53, p21, and p16, enhanced proliferation, migration, adhesion, and secretion of EPCs	[59]
hAD-MSCs	Artemisia argyi water extract	Doxorubicin-induced models	Upregulation of Klotho/TERT, elongating telomeres and reducing mitochondrial superoxide	[60]
WJ-MSCs	Ohwia caudata aqueous extract	Doxorubicin-induced models	Restoration of mitochondrial function, reduction of ROS, stabilization of membrane potential, and decreasing apoptosis	[61]
ISCs	Total ginsenosides	Elderly drosophila	Inhibition of IRE1-JNK pro-apoptotic UPR pathway	[62]

Abbreviations: CM, Chinse medicine; HSCs, hematopoietic stem cells; BMSCs, bone marrow mesenchymal stem cells; OGSCs, ovarine germline stem cells; EpiSCs, Epidermal stem cells; EPCs, endothelial progenitor cells; hAD-MSCs, human adipose derived-mesenchymal stem cell; WJ-MSCs, Wharton’s jelly-derived mesenchymal stem cells; ISCs, intestinal stem cells; YAP, Yes-asssociated protein; TERT, telomerase reverse transcriptase; ROS, reactive oxygen species

## CM promotes stem cell proliferation and differentiation

Stem cell proliferation [65] and differentiation [66] are essential processses for tissue repair and regeneration. Accumulating evidence indicates that CM promotes stem cell proliferation and differentiation across diverse pathological conditions, including inflammatory bowel disease (IBD) [29, 67], sleep disorders [68], knee osteoarthritis [69], diabetes [70], and neurodegenearive diseases [71]. Additionaly, CM enhances these process within damaged tissues, accelerating healing [72]. These beneficial effects stem from CM’s ability to modulate multiple signaling pathways [73–78].

The Wnt signaling pathway comprises intracellular transduction cascades initiated by Wnt ligand binding. In the canonical Wnt/β-catenin pathway, Wnt binding to frizzled (Fz) receptors and low-density lipoprotein receptor-related protein 5/6 (LRP5/6) co-receptors triggers the formation of a receptor complex, which activates dishevelled (Dvl) proteins. Dvl inhibits the β-catenin destruction complex, preventing β-catenin in degradation. Consequently, β-catenin accumulates in the cytoplasma, translocates to nucleus, and interacts with transcription factors to activate Wnt target genes regulating critical cellular functions like proliferation and differentiation [79, 80]. In chronic atrophic gadtritis (CAG) rat models, low Wnt pathway activation inhibits gastric stem cell (GSC) proliferation and differentiation, impairing mucosal repair. Jianpi Yiqi formula (健脾益气方) counteracted this by enhancing Wnt3a expression and upregulating GSC markers (e.g., Lgr5, Sox2, Ki67, PCNA, and Muc5A), ameliorating atrophy [81]. The miR-217/RUNX2 axis regulates downstream osteogenic markers ALP and OPN and modulates Wnt/β-catenin signaling. Shuanglongjiegu pill (双龙解骨片) promoted BMSC osteogenic differentiation by modulating this axis and activating the Wnt/β-catenin [82]. Similarly, Dahuang Gancao decoction (大黄甘草汤) activated and proliferated HFSCs via this pathway [83]. Stromal cell-secreted Wnt ligands within the ISC niche regulate ISC proliferation and differentiation. In colitis models, berberine promoted Wnt expression in colorectal stromal cells, optimizing the niche and enhancing mucosal repair, an effect diminished by inhibiting stromal Wnt secretion [84]. Wnt signaling induces β-catenin nuclear translocation, activating target genes (e.g., c-Myc and Cyclin D1) to promote hepatocyte progenitor cell differentiation and maturation. Sinisan (四逆散) activated Wnt signaling in liver injury models, accelerating this process [85]. Ginseng suppressed glycogen synthase kinase 3β (GSK-3β) expression in aging models, leading to β-catenin accumulation and increased expression of Wnt targets (e.g., Lgr5 and Olfm), supporting ISC proliferation and differentiation [86, 87]. Oleanolic acid (OA), inhibited GSK-3β activity (via Ser9 phosphorylation), stabilizing β-catenin and promoting its nuclear translocation to drive NSC proliferation and differentiation [88]. Jujuboside A (JA) directly upregulated Wnt3a and β-catenin, increasing expression of genes critical for amyloid precursor protein-overexpressing neural stem cells (APP-NSCs) proliferation and differentiation [89]. Icariin II, inhibited apoptosis, enhances viability, increases phosphorylated GSK-3β (p-GSK-3β), and induces β-catenin nuclear translocation in APP-NSCs [90]. These findings underscore the critical role of Wnt/β-catenin signaling in stem cell-mediated regeneration and CM’s capacity to modulate it [91]. The effectiveness of CM in regulating this pathway suggests synergy when combined with stem cell therapies, offering promising avenues for regenerative medicine [15, 92, 93].

The PI3K/AKT signaling hub regulates fundamental cellular processes (e.g., proliferation, differentiation, and apoptosis), which is crucial for stem cell function [94, 95]. The n-butanol extract of Gualou Xiebai Banxia decoction (瓜蒌薤白半夏汤) [96] and Astragalus (黄芪) [97] enhanced PI3K/AKT expression and phosphorylation, counteracting hypoxia-ischemia-induced apoptosis in BMSCs and improving viability and tolerance. Guishen Wan (归肾丸) aqueous extract promoted BMSC proliferation by upregulating key PI3K/AKT pathway proteins [98]. Baoyuan Capsule (保元胶囊) enhanced NSC differentiation and neural repair by increasing AKT and phosphorylated (p-AKT) expression, ameliorating functional deficits in cerebral ischamis-hypoxial models [99]. Yangjing capsule (养精胶囊) drove spermatogonial stem cell (SSC) proliferation via PI3K/AKT pathway activation and downstream Cyclin D1 induction, increasing S-phase cell proportion [100]. This mechanism extends to ISCs [101], human periodontal ligament stem cells (hPDLSCs) [102]. Ginsenoside Rg1 exerted antioxidant autophagic, and proliferative effects, primarily via AKT and its downstream target mammalian target of rapamycin (mTOR) activation [103]. Thus, PI3K/AKT pathway modulation represents a central convergent mechanism for diverse CM formulations to enhance stem cell functionality across sources [104, 105]. Future research priorities include determining the specificity of CM modulation within this complex network and validating therapeutic safety or efficacy in preclinical and clinical settings.

Robust evidence positions MAPK/ERK signaling as pivotal mechanistic convergence points for CM compounds regulating stem cell proliferation, differentiation, and functional restoration in neorological, musculoskeletal, and gastrointestinal contexts [106, 107]. Compounds like Astragaloside IV [108], Asperosaponin VI [109], PEEPA-P5 [110], berberine [111], and naringin [112] activated phosphorylated nodes (e.g., p-EGFR, p-MAPK, p-ERK1/2), achieving therapeutic outcomes from neurogenesis to osteoporosis. Phosphorylated MAPK (p-MAPK) transduces signals to ERK. Subsequently, p-ERK translocates to nucleus, regulating transcription factors and mediating celluler functions. Notably, the key limitations exists. Over-reliance on rodent models with limited validation in human cells or clinical cohorts. Besides limited quantification of CM bioavailability and ERK activation dynamics hinders understanding of optimal dosing and potential off-terget effects.

While the Wnt/β-catenin, PI3K/AKT and MAPK/ERK pathways are individually crucial role for proliferation and differentiation, they exhibit significant crosstalk [113]. CM’s inherent multi-target regualtion advantage enables simultaneous modulation of these interconnected pathways, promoting stem cell-based tissue repair [71, 114]. Integrating CM, with its multi-pathway regulatory capacity, with modern stem cell approaches holds significant potential for treating diverse tisue injuries, refractory diseases, and rare disorders in regenerative medicine. Key findings of CM enhancing stem cell proliferation and differentiation are summarized in Table 3.

**Table 3:** CM promotion on stem cell proliferation and differentiation

Cell type	CM	Diseases/Models	Effects	References
GSCs	Jianpi Yiqi formula (健脾益气方)	Chronic atrophic gastritis rat models	Elevated levels of Wnt3a and GSC markers	[81]
BMSCs	Shuanglongjiegu pill (双龙解骨片)	Rat BMSCs	activation of Wnt/β-catenin pathway	[82]
HFSCs	Dahuang Gancao decoction (大黄甘草汤)	Androgenetic alopecia (AGA) mouse models	Activation of Wnt/β-catenin pathway	[83]
ISCs	Berberine	Colitis mice models	Promotion of Wnt expression, optimizing the niche and enhancing mucosal repair	[84]
Hepatic stem cell	Sinisan (四逆散)	Liver injury mice models	Activation of Wnt signaling	[85]
ISCs	Ginseng	Aging mouse models	Suppression of glycogen synthase kinase 3β (GSK-3β)	[86, 87]
NSCs	Oleanolic acid (OA)	Rat subventricular zone	Inhibition of GSK-3β activity, stabilizing β-catenin and promoting its nuclear translocation	[88]
APP-NSCs	Jujuboside A (JA)	AD mice models	Upregulation of Wnt3a and β-catenin	[89]
APP-NSCs	Icariin II	Cell experiment	Inhibition of apoptosis, enhanced Cell viability, elevated p-GSK-3β levels	[90]
BMSCs	Gualou Xiebai Banxia decoction (瓜蒌薤白半夏汤)	Cell experiment	Enhanced PI3K/AKT expression and phosphorylation	[96]
BMSCs	Astragalus membranaceu (黄芪)	Cell experiment	Enhanced PI3K/AKT expression and phosphorylation	[97]
BMSCs	Guishen Wan (归肾丸)	Cell experiment	Upregulation of key PI3K/AKT pathway proteins	[98]
NSCs	Baoyuan Capsule (保元胶囊)	Transient middle cerebral artery occlusion (MCAO) ischemic mice models	Increased expression of AKT and p-AKT	[99]
SSCs	Yangjing capsule (养精胶囊)	Cell experiment	Activation of PI3K/AKT pathway and downstream Cyclin D1 induction	[100]
ISCs	Paeoniflorin	UC mice models	Activation of PI3K/AKT pathway	[101]
hPDLSCs	Berberine	Cell experiment	Activation of PI3K/AKT pathway	[102]
BMSCs	Ginsenoside Rg1	Cell experiment	Activation of AKT and mammalian target of rapamycin (mTOR)	[103]
NSCs	Astragaloside IV,	Transient cerebral ischemic rat models	Upregulated expression of phosphorylated epidermal growth factor receptor (p-EGFR), p-MAPK, and ERK1/2	[108]
hMSCs	Asperosaponin VI	Cerebral Ischemia-Reperfusion rat models	Activation of p-ERK 1/2	[109]
GSCs	PEEPA-P5	Chronic atrophic gastritis mouse models	Increase expression of p-ERK1/2	[110]
ISCs	Berberine	Radiation-injured mice models	suppress the apoptosis of crypt epithelial cells, increased quantity of goblet cells, and increased quantity of OLFM4^+^ ISCs and tdTomato^+^ progenies	[111]
BMSCs	Naringin	Ovariectomized rat models	Increase expression of osteocalcin	[112]

Abbreviations: CM, Chinse medicine; GSCs, gastric stem cells; BMSCs, bone marrow mesenchymal stem cells; HFSCs, hair follicle stem cells; ISCs, intestinal stem cells; APP-NSCs, precursor protein-overexpressing neural stem cells; SSCs, spermatogonial stem cells; hPDLSCs, human periodontal ligament stem cells; NSCs, neural stem cells

## CM stimulates stem cell homing, engraftment and survival

Stem cell homing refer to the coordinated migration of stem cells through the peripheral circulation towards specific target organs. This process is guided by chemokines, cytokines, growth factors, adhesion molecules, and enzymes released from injuried tissues, which bind corresponding receptors on stem cells, directing their mobilization, migration, retention, and engraftment [115, 116]. Mobilization and migration involve complex molecular cascades [117], with stem cell factor (SCF) being a key mobilizing agent [118, 119]. Studies have demonstrated that CM preparations enhance homing mechanisms. The CM formulations Xuesaitong (血塞通) [120] and modified Taohong Siwu decoction [121] promoted endogenous stem cell mobilization in rat model of cerebral infraction and myocardial ischemia, respectively, by elevating SCF levels in plasma, bone marrow, and serum. Further research is needed to confirm concurrent upregulation of downstream SCF receptors. Stromal cell-derived factor 1 (SDF-1) is crucial for recruitment stem cell (e.g., cardiac stem cells) and facilitating repair post-injury [122]. Tissue injury locally elevated SDF-1, creating a chemotactic gradient that activate its cognate receptor CXCR4 on stem cells, driving transendothelial migration to the damage site. This mechanism is exploited by CM formulations. The Chuangxiong (川芎)-Chishao (赤芍) herb pair enhanced SDF-1 driven angiogenesis in ischemic myocardium [123]. Bushen Huoxue recipe (补肾活血方) [124] and Astragalus polysaccharides [125], promoted migration of exogenous BMSCs to ameliorate tissue injury via SDF/CXCR4 axis. Beside, successful therapeutical outcomes depend critically on stem cell retention, engrftment (seeding) and survival within the target tissue. Retention and engraftment are significantly regulated by the SDF-1/CXCR4 axis too [126, 127]. Danhong injection (丹红注射液) boosted CXCR4 expression on MSCs and SDF-1 levels in myocardium, significantly enhancing MSC retention in cardiac tissue [128]. Similarly, Guanxin Danshen formulation (冠心丹参方) synergizes with MSCs to improve outcomes in ischemic injury by upregulating infract-zone SDF-1, reducing apoptosis, and enhancing angiogenesis beyond monotherapy effects [129]. Poor cell survival and engraftment remain major limitations for stem cell therapy [130]. CM enhances these processes. Resveratrol, derived from Huzhang (虎杖), significantly improved the survival and engraftment of hUC-MSCs in the hippocampal region of Alzheimer’s disease (AD) model mice [131]. This leads to improved learing and memory, and reduced neuronal apoptosis. CM formulations can mitigate complications like graft-versus-host disease (GVHD), a common and serious tissue following allogeneic hematopoietic stem cell transplantaion and impacts transplanted cell survival [132]. TGF-β1 and BMPs promote cell survival by activating NF-κB signaling. TGF-β1 binding triggers a cascade culminating in NF-κB nuclear translocation and the expression of pro-survival and anti-apoptotic genes [133]. BMPs, similarly modulate NF-κB through non-canonical pathways to exert anti-apoptotic effects in various cell types [134]. Safely elevated doses of Danzikang knee granule (丹子康膝颗粒) were positively correlated with increased BMSCs survival rates. This effects were associated with significantly elevated serum levels of TGF-β1, BMP2 and BMP4 [69].

While stem cell therapy holds immense promise for regenerative medicine due to its inherent tissue regenerative capacity, clinical efficacy is often hampered by limited homing, poor survival, and insufficient retentiona and engraftment of administered cells [135]. Substantial evidence confirms that CM effectively improve stem-cell based therapeutical outcomes by optimizing these key processes post-transplantation. The relevant research has been summarized in Table 4.

**Table 4:** CM stimulation on stem cell homing, engraftment and survival

Cell type	CM	Diseases/Models	Effects	References
Endogenous stem cell	Chinese preparation Xuesaitong (血塞通)	Cerebral infarction rat models	Elevating SCF levels in bone marrow and mobilization of BMSCs	[120]
Endogenous stem cell	Modified Taohong Siwu decoction (桃红四物汤加减)	I/R rat models	Elevating SCF and SDF-1 levels	[121]
Cardiomyocytes	Chuangxiong (川芎) and Chishao (赤芍)	Myocardial infarction (MI) mouse models	Enhanced SDF-1 and mobilization of stem cells	[123]
BMSCs	Bushen Huoxue recipe (补肾活血方)	Premature ovarian insufficiency mouse models	PromotIion of migration of BMSCs via SDF/CXCR4 axis	[124]
BMSCs	Astragalus polysaccharides	Animal experiment	Promotion of migration of BMSCs via SDF/CXCR4 axis	[125]
MSCs	Danhong injection (丹红注射液)	Myocardial infarction (MI) mice models	Increased CXCR4 expression	[128]
MSCs	Guanxin Danshen formulation (冠心丹参方)	Myocardial infarction (MI) rat models	Upregulating infract-zone SDF-1, reducing apoptosis, and enhancing angiogenesis	[129]
hUC-MSC	Resveratrol	Alzheimer’s disease (AD) mice models	Improved the survival and engraftment	[131]
BMSCs	Danzikang knee granule (丹子康膝颗粒)	Animal experiemnts	Improved survival rates	[69]

Abbreviations: CM, Chinse medicine; BMSCs, bone marrow mesenchymal stem cells; MSCs, mesenchymal stem cells; hUCMSCs, human umbilical cord mesenchymal stem cells; SCF, stem cell factor; SDF-1, stromal cell-derived factor 1

## CM boosts stem cell-derived exosomes secretion

Stem cell-derived exosomes (SC-Exos) are small vesicles (30 ~ 150 nm) released via exocytosis. They encapsulate bioactive molecules, including proteins, lipids, mRNAs, and miRNAs, and mediate intercellular communication, playing crucial roles in signal transduction [136], tissue repair [137], and immune regulation [138]. Their advantageous properties, including high biocompatibility, stability, low toxicity, and efficient cargo transfer, make SC-Exos excellent candidates for regenerative medicine and tissue engineering [139]. Consequently, SC-Exos hold significant potential for treating neurological [140], gastrointestinal [141], and joint diseases [142].

Emerging evidence indicates that CM enhances the secretion of SC-Exos and modifies their molecular cargo, thereby exerting protective effects on cardiovascular, nervous musculoskeletal, and other system. Post-MI damaged cardiomyocytes release DAMPs, activating TLR4 signaling on cardiac macrophages and cardiomyocytes. This recruits My88, leading to phosphorylation of interleukin-1 receptor-associated kinase 2 (IRAK2). IRAK2’s extended half-life enables sustained signaling, promoting NF-κB p65 nuclear translocation, cytokine storms, adverse ventricular remodeling, immune infiltration, and cardiomyocyte death. Reducing IRAK2 expression suppresses NF-κB activity and infarct size [143]. Preconditioning MSCs with Tongxinluo (通心络) enhanced secretion of exosomes enriched in miRNA-146a-5p [144]. This miRNA targets and downregulates IRAK2 expression, inhibiting NF-κB p65 nuclear translocation and protecting cardiomyocytes from hypoxic injury. C-C Chemokine Receptor Type 2 (CCR2) activation promotes immune cell infiltration, amplifies inflammation, and drives cardiomyocyte death. Tanshinone IIA treatment increased miRNA-223-5p levels in MSC-derived exosomes [145]. These exosomes alleviate I/R injury by inhibiting CCR2 activation, reducing monocyte infiltration, and enhancing angiogenesis. Preconditioning NSCs with Lycium barbarum polysaccharide increased NSC-Exo secretion and enriched them with miRNA-133a-3p [146]. This miRNA activates the AMPK/mTOR pathway, inhibiting stroke-induced autophagy and potentially mitigating neuronal damage. The precise temporal window of autophagy inhibition requires further elucidation. Catalpol, a bioactive compound from Rehmannia glutinosa, promoted NSC secretion of exosomes enriched in miRNA-138-5p [147]. These exosomes improve neural development and survival, slowing AD progress. Psoralen, derived from Psoralea corylifolia, significantly altered the miRNA profile (93 miRNA differentially expressed) in exosomes secreted by hPDLSCs [148]. Notably downregulation of hsa-miRNA-125b-5p promotes osteogenic differentiation, contributing to periodontal tissue regeneration.

Although research on CM’s promotion of SC-Exo secretion is still developing, current evidence demonstrate that CM acting as exosome modulators exerts beneficial effects across multiple disorders, primarily by influencing inflammatory pathways and tissue damage responses via enhanced or modified SC-Exo secretion. So, the future research should focus on elucidating the broader spectrum of CM effects on SC-Exo cargo and secretion mechanisam, defining the optimal temporal windows for interventions (e.g., autophagy modulation in stroke), exploring the therapeutical synergy between CM and SC-Exos in greater depth. CM promotes SC-Exo secretion is presented in Table 5.

**Table 5:** CM boosts stem cell-derived exosomes secretion

Cell type	CM	Dideases/Models	Effects	References
MSCs	Tongxinluo (通心络)	Acute myocardial infarction (AMI) rat models	Induction of miRNA-146a-5p secretion	[144]
MSCs	Tanshinone IIA	Myocardial I/R injury rat models	Elevation of miRNA-223-5p levels	[145]
NSCs	Lycium barbarum polysaccharide	Middle cerebral artery occlusion mice models	Enrichment of miRNA-133a-3p	[146]
NSCs	Catalpol	AD mice models	Promotion of miRNA-138-5p secretion	[147]
hPDLSCs	Psoralen	Cell experiment	Downregulation of hsa-miRNA-125b-5p	[148]

Abbreviations: CM, Chinese Medicine; MSCs, mesenchymal stem cells; NSCs, intestinal stem cells; hPDLSCs, human periodonntal ligament stem cells; AD, Alzheimer’s disease

## Additional regulatory effects of CM on stem cells

Beyound the previously discussed mechanism, CM exerts multifaceted regulatory effects on stem cells. These include enhancing blood perfusion, modulating gene expression, influencing metabolic pathways, and regulating intercellular interactions. CM’s accessibility and multi-target nature make it a valuable adjunct to stem cell-based therapies. Adequate blood supply is critical for stem cell survival and function. CM promotes angiogenesis, improving local perfusion and supporting transplanted stem cells. For instance, in ischemic stroke models, specific CM formulations significantly increased microvessel density (measured by CD31-positive staining) surrounding transplanted bone marrow-derived BMSCs, correlating with improved functinoal outcomes [149]. CM can directly influence gene expression profiles in stem cells and progenitor cells. Total flavonoids from Litchi chinensis inhibited the expression and nuclear translocation of Notch3 in breast cancer stem cells (BCSCs) [150, 151]. This downregulates key transcription factors Hes1 and Runx2, utimately reducing oncogene expression and potentially mitigating and tumorigenicity risk associated with stem cell therapies. Further research is warranted to fully validate this anti-tumorigenic potential. Cellular metabolism profoundly impacts stem cell quiescence, activation, and function. CM can modulate key metabolic pathways. Spermidine, a vital polyamine metabolite, declines with age and is essential for cell growth, proliferation, and genomic stability. Crucially, spermidine serves as the substrate for hypusination, a post-translational modification critial for activating eukaryotic translation initiation factor 5 A (elF5A). Activated elF5A is vital for transitioning quiescent stem cells to proliferative state, enabling regeneration. Ginseng extract elevated spermidine levels in aged mice, promoting elF5A hypusination and activating skeletal muscle stem cells, thereby facilitating muscle regeneration [152, 153]. HIF-1αand ERK1/2 are central regulator of cellular metabolism, influencing metabolic enzymes, transporters, and gene expression to orchestrate energy production and biosynthesis. BuyangHuangwu Decoction (补阳还五汤) alleviated endothelial cell apoptosis, ensuring sustained HIF-1α secretion. HIF-1α triggers the ERK1/2 signaling cascade, stimulating osteogenic differentiation in MSCs and promoting bone regeneration [154, 155]. By modulating signaling molecules like HIF-1α, CM indirectly influences the complex network of intercellular communication within stem cell niches, impacting cell-cell signaling and nice-stem cell crosstalk.

CM’s ability to simultaneously target multiple aspects of stem cell biology, including perfusion, gene expression, metabolism, and intercellular signaling, underprint its significant potential as a regulator to optimize stem cell-based therapies. This multi-target action is a key advantage of CM in regenerative medicine. Additional regulatory effects of CM on stem cells is shown in Table 6.

**Table 6:** Additional regulatory effects of CM on stem cells

Cell type	CM	Effects	References
BCSCs	Total flavonoids	Downregulation of factors Hes1 and Runx2	[150, 151]
Skeletal muscle stem cells	Ginseng extract	Promoting of elF5A hypusination	[152, 153]
MSCs	BuyangHuangwu Decoction (补阳还五汤)	Sustained HIF-1α secretion	[154, 155]

Abbreviations: CM, Chinese Medicine; BCSCs, breast cancer stem cells; MSCs, mesenchymal stem cells; elF5A, eukaryotic translation initiation factor 5 A; HIF-1α, hypoxia-inducible fator 1α

## Challenges and future prospects

CM, with its millennia of dicumented clinical efficacy, and stem cell therapy, representing cutting-edge regenerative potential, together offer a promising frontier for innovative treatments. However, significant challenges must be addressed to realizes their synergistic potential. Firstly, theorectical and methodological disparities. Fundamental differences exist between TCM’s personalized paradigm, characterized by syndrome differentiation and customized prescriptions (‘one person, one formula’), and the standardized protocols of stem cell therapy (precise cell types, dosage, and delivery routes). This divergence complicates direct comparisons in clinical trials and impedes systematic validation of combined efficacy. Bridging these distinct therapeutic philosophies requires novel framework for integration. Secondly, interdisciplinary collaboration gaps. The convergence of TCM and stem cell biology demands deep expertise in both domains. Currently, a critical shortage of researchers proficient in both fields hinders effective collaboration. Overcoming conceptual barriers and fostering truly interdisciplinary teams is essential for translating basic research into clinically viable strategies. Thirdly, translational discrepancies in research models. Current mechanistic research primarily investigates TCM compounds’ effects on isolated stem cells in vitro. Far fewer studies probe these interactions within these complex in vivo microenvironment. Preclinical models often use localized stem cell delivery, while clinical practice favors systemic intravenous infusion. This methodological disconnecy potentially alters stem cell efficact and obscures how CM might enhance systemic delivery or survival. Consequently, promising results from animal studies lack robust validation in human trials. Subsequent research must be strategically guided by target conditions where both CM and stem cells show mechanistic relevance, leveraging CM’s strength in dynamic disease stating and optimizing cell type and delivery strategies for specific pathologies.

Despite these hurdles, the dynamic nature of diseases presents a compelling rationale for combining these modalities. CM’s ability to adapt interventions to specific disease stages offers a unique to enhance stem cell therapy, potentially improving homing, survival, or function at critical points in the treatment timeline. While current clinical evidence remains limited, a concerted focus on bridging theorectical, interdisciplinary, and translational gaps will pave the way for validated, synergistic therapies.

## Conclusion

Stem cell therapy holds significan promise for treating a wide spectrum of diseases and injuries. CM offers substential potential to optimize this approach by modulating the stem cell environmen, delaying senescence, promoting proliferation and differentiation, facilitating homing and engraftment, enhancing survival, stimulating exosomes secretion, and exerting other beneficial effects. The strategic integration of CM with stem cell therapy therefore represents a compelling approach to improve treatment efficacy for various refractory conditions. While significant challenges remain, ongoing research provides encouraging progress. Future efforts must prioritize optimizing treatment protocols and conducting rigorous clinical validation to firmly establish the therapeutical advantages of this synergistic combination.

## Data Availability

Not applicable.

## References

[1] Anthony Atala S, Murphy. Regenerative Medicine. JAMA. 2015;313(14):1413–4. 10.1001/jama.2015.1492 25871665

[2] Swijnenburg R-J, Schrepfer S, Cao F, Pearl JI, Xie X, Connolly AJ, et al. JC wu: in vivo imaging of embryonic stem cells reveals patterns of survival and immune rejection following transplantation. Stem Cells Dev. 2008;17(6):1023–9. 18491958 10.1089/scd.2008.0091 PMC2657199

[3] Xinrong Peng T, Liu Y, Wang Q, Jin YH, Li L, et al. Wu: Wnt/beta-catenin signaling in embryonic stem cell converted tumor cells. J Translational Med. 2012;10(196):1–9. 10.1186/1479-5876-10-196 PMC3515512 22995718

[4] Li Li C, Jianfei S, Yanhong. Modeling neurological diseases using iPSC-derived neural cells. Cell Tissue Res. 2017;371(1):143–51. 29079884 10.1007/s00441-017-2713-x PMC6029980

[5] Lin Y, Sato N, Hong S, Nakamura K, Ferrante EA, Yu ZX, Dunbar CE, et al. Long-term engraftment and maturation of autologous iPSC-derived cardiomyocytes in two rhesus macaques. Cell Stem Cell. 2024;31(7):974–e988975. 38843830 10.1016/j.stem.2024.05.005 PMC11227404

[6] Sukegawa M, Miyagawa Y, Kuroda S, Yamazaki Y, Yamamoto M, Adachi K, et al. Okada: mesenchymal stem cell origin contributes to the antitumor effect of oncolytic virus carriers. Mol Therapy: Oncol. 2024;32(4):1–17. 10.1016/j.omton.2024.200896 PMC11568361 39554905

[7] Meenu Kalkal M, Tiwari RS, Thakur V, Awasthi V, Pande D, Chattopadhyay J, Das. Mesenchymal stem cells: a novel therapeutic approach to enhance protective immunomodulation and erythropoietic recovery in malaria. Stem Cell Reviews Rep. 2021;17(6):1993–2002. 10.1007/s12015-021-10191-1 PMC8196918 34117997

[8] Barker N. Adult intestinal stem cells: critical drivers of epithelial homeostasis and regeneration. Nat Rev Mol Cell Biol. 2014;15(1):19–33. 24326621 10.1038/nrm3721

[9] Peterson A, Nair LS. Hair follicle stem cells for tissue regeneration. Tissue Eng Part B: Reviews. 2022;28(4):695–706. 10.1089/ten.teb.2021.0098 PMC9419938 34238037

[10] Culig L, Chu X, Bohr VA. Neurogenesis in aging and age-related neurodegenerative diseases. Ageing Res Rev. 2022;78:1–66. 10.1016/j.arr.2022.101636 PMC9168971 35490966

[11] Poetsch MS, Strano A, Guan K. Human induced pluripotent stem cells: from cell origin, genomic stability, and epigenetic memory to translational medicine. Stem Cells. 2022;40(6):546–55. 35291013 10.1093/stmcls/sxac020 PMC9216482

[12] Li Z, Liang Y, Wang Y, Lin Y, Zeng L, Zhang Y, Zhu L. Zuogui pills alleviate cyclophosphamide-induced ovarian aging by reducing oxidative stress and restoring the stemness of oogonial stem cells through the Nrf2/HO-1 signaling pathway. J Ethnopharmacol. 2024;333:1–17. 10.1016/j.jep.2024.118505 38945466

[13] Zhang Lili G, Hao C, Sifang R, Xingde W, Yun. State-regulating medicine: an integration of traditional Chinese medicine and biomedicine. Zhongguo Zhong Yao Za Zhi. 2021;46(16):4300–6. 34467745 10.19540/j.cnki.cjcmm.20210510.601

[14] Li H-M. Clinical trial with traditional Chinese medicine intervention ‘’tonifying the kidney to promote liver regeneration and repair by affecting stem cells and their microenvironment’’ for chronic hepatitis B-associated liver failure. World J Gastroenterol. 2014;20(48):18458–66. 25561817 10.3748/wjg.v20.i48.18458 PMC4277987

[15] Ma Y, Bao Y, Wang H, Jiang H, Zhou L, Yang B, et al. Zhang: 1H-NMR-based metabolomics to dissect the traditional Chinese medicine promotes mesenchymal stem cell homing as intervention in liver fibrosis in mouse model of wilson’s disease. J Pharm Pharmacol. 2024;76(6):656–71. 38429940 10.1093/jpp/rgae016

[16] Zhang Xin C, Jingjing Z, Jing Z, Zhichen. The effect of Bushen Huazhuo formula on improving oxidative stress indicators in patients with vascular dementia after stroke. Chin Med. 2022;(7):212–4.

[17] Xiang Lu Z, Qiaoyan Z, Qiming Q, Luping G, Wan. Research progress on chemical constituents, pharmacological effects and clinical applications of astragali radix-angelicae sinensis radix. Chin Traditional Herb Drugs. 2022;53(7):2196–213.

[18] Wang Yunbao Z, Juan C, Ying HM, Xie W, Daojun X, Xiaofeng H. Liver regeneration effect and mechanism of Bushen Huoxue Huazhuo recipe on Wilson disease liver fibrosis of TX mice. China J Traditional Chin Med Pharm. 2022;37(1):159–64.

[19] Ziqiao Y, Li Y, Xia T, Wang K, Liao Z, Zhang L, Gao Y, et al. Revitalizing gut health: Liangxue Guyuan Yishen Decoction promotes Akkermansia muciniphila -induced intestinal stem cell recovery post-radiation in mice. Phytomedicine. 2024;132:1–15. 10.1016/j.phymed.2024.155888 39084128

[20] Yu Wang Y, Xue H, Guo. Intervention effects of traditional Chinese medicine on stem cell therapy of myocardial infarction. Front Pharmacol. 2022;13:01–16. 10.3389/fphar.2022.1013740 PMC9622800 36330092

[21] Du H, Zhang T, Wang Q, Cao X, Zheng H, Li J, et al. Y sun: traditional Chinese medicine Shi-Bi-Man regulates lactic acid metabolism and drives hair follicle stem cell activation to promote hair regeneration. Chin Med. 2023;18(84):1–15. 37454125 10.1186/s13020-023-00791-z PMC10349503

[22] Xu Z, Gong B, Li Z, Wang Y, Zhao Z, Xie L, et al. Y bian: Bazi Bushen alleviates skin senescence by orchestrating skin homeostasis in SAMP6 mice. J Cell Mol Med. 2023;27(18):2651–60. 37614114 10.1111/jcmm.17833 PMC10494291

[23] Dietrich KS-DP, Wu H-F, Xin S, Patel AJ, Camryn Gale Wzient eket al. N Zeltner: genipin crosslinks the extracellular matrix to rescue developmental and degenerative defects, and accelerates regeneration of peripheral neurons. bioRxiv. 2023;03.22.533831.

[24] Guo X, Ma R, Lau BW-M, Wang M, Chen X, Li Y. Novel perspectives on the therapeutic role of cryptotanshinone in the management of stem cell behaviors for high-incidence diseases. Front Pharmacol. 2022;13:01–14. 10.3389/fphar.2022.971444 PMC9420941 36046823

[25] Congzi Wu Z, Shi Q, Ge H, Xu Z, Wu P, Tong H, Jin. Catalpol promotes articular cartilage repair by enhancing the recruitment of endogenous mesenchymal stem cells. J Cell Mol Med. 2024;28(7):1–12. 10.1111/jcmm.18242 PMC10955160 38509736

[26] Wei Q, Hao X, Lau BW-M, Wang S, Li Y. Baicalin regulates stem cells as a creative point in the treatment of climacteric syndrome. Front Pharmacol. 2022;13:01–13. 10.3389/fphar.2022.986436 PMC9666758 36408261

[27] Fu J, Xie X, Yao H, Xiao H, Li Z, Wang Z, Zhang N, et al. The effectiveness of traditional Chinese medicine in treating malignancies via regulatory cell death pathways and the tumor immune microenvironment: a review of recent advances. Am J Chin Med. 2024;52(1):137–60. 38328830 10.1142/S0192415X2450006X

[28] Yu Z-l, Gao R-y, Lv C, Geng X-l, Ren Y-j, Zhang J, Dou W, et al. Notoginsenoside R1 promotes Lgr5 + stem cell and epithelium renovation in colitis mice via activating Wnt/β-Catenin signaling. Acta Pharmacol Sin. 2024;45(7):1451–65. 38491161 10.1038/s41401-024-01250-7 PMC11192909

[29] Shuguang Y, Wang P, Wei H, Jia R, Zhen M, Li Q, Li J, et al. Treatment of ulcerative colitis with Wu-Mei-Wan by inhibiting intestinal inflammatory response and repairing damaged intestinal mucosa. Phytomedicine. 2022;105:1–13. 10.1016/j.phymed.2022.154362 35947900

[30] Hicks MR, Pyle AD. The emergence of the stem cell niche. Trends Cell Biol. 2023;33(2):112–23. 35934562 10.1016/j.tcb.2022.07.003 PMC9868094

[31] Clevers H, Loh KM, Nusse R. An integral program for tissue renewal and regeneration: Wnt signaling and stem cell control. Science. 2014;346(6205):5451–57. 10.1126/science.1248012 25278615

[32] Zhao B-B, Li H-M, Gao X, Ye Z-H, Cheng S-S. The herbal compound Diwu Yanggan modulates liver regeneration by affecting the hepatic stem cell microenvironment in 2-acetylaminofluorene/partial hepatectomy rats. Evidence-Based Complementary and Alternative Medicine. 2015;2015:1–7. 10.1155/2015/468303 PMC4299675 25628749

[33] Tsai ST, Nithiyanantham S, Satyanarayanan SK, Su KP. Anti-inflammatory effect of traditional Chinese medicine on the concept of mind-body interface. Adv Exp Med Biol. 2023;1411:435–58. 36949321 10.1007/978-981-19-7376-5_19

[34] Zhang L, Wei W. Anti-inflammatory and immunoregulatory effects of paeoniflorin and total glucosides of paeony. Pharmacol Ther. 2020;207(107452):1–12. 10.1016/j.pharmthera.2019.107452 31836457

[35] Sirish P, Thai PN, Lee JH, Yang J, Zhang X-D, Ren L, Chiamvimonvat N, et al. Suppression of inflammation and fibrosis using soluble epoxide hydrolase inhibitors enhances cardiac stem cell-based therapy. Stem Cells Translational Med. 2020;9(12):1570–84. 10.1002/sctm.20-0143 PMC7695637 32790136

[36] Fu Y, Kong Y, Li J, Wang Y, Li M, Wang Y, Chang Z, et al. Mesenchymal stem cells combined with traditional Chinese medicine (qi-fang‐bi‐min‐tang) alleviates rodent allergic rhinitis. J Cell Biochem. 2019;121(2):1541–51. 31535402 10.1002/jcb.29389

[37] Li Y, Huang J, Wang J, Xia S, Ran H, Gao L, Yuan J, et al. Human umbilical cord-derived mesenchymal stem cell transplantation supplemented with curcumin improves the outcomes of ischemic stroke via AKT/GSK-3β/β-TrCP/Nrf2 axis. J Neuroinflamm. 2023;20(1):1–23. 10.1186/s12974-023-02738-5 PMC9951499 36829224

[38] Mesenchymal stem cell. Response to growth factor treatment and low oxygen tension in 3-dimensional construct environment. Muscles Ligaments Tendons J. 2014;4(1):46–51. 24932447 PMC4049650

[39] Tao Qi H, Gao Y, Dang S, Huang M, Peng. Cervus and cucumis peptides combined umbilical cord mesenchymal stem cells therapy for rheumatoid arthritis. Medicine. 2020;99(28):1–6. 10.1097/MD.0000000000021222 PMC7360298 32664175

[40] Mao Y, Meng L, Liu H, Lu Y, Yang K, Ouyang G, Chen S, et al. Therapeutic potential of traditional Chinese medicine for vascular endothelial growth factor. J Zhejiang University-SCIENCE B. 2022;23(5):353–64. 10.1631/jzus.B2101055 PMC9110323 35557037

[41] Guo JW, Chen C, Huang Y, Li B. Combinatorial effects of Naomai Yihao capsules and vascular endothelial growth factor gene-transfected bone marrow mesenchymal stem cells on angiogenesis in cerebral ischemic tissues in rats. J Tradit Chin Med. 2012;32(1):87–92. 22594109 10.1016/s0254-6272(12)60038-7

[42] Zhirong Luo W, Meng H, Li Y, Wang Y, Wang Y, Zhao H, Guo, et al. Transplantation of induced pluripotent stem cells-derived cardiomyocytes combined with modified Taohong Siwu Decoction improved heart repair after myocardial infarction. Heliyon. 2024;10(4):1–12. 10.1016/j.heliyon.2024.e26700 PMC10906439 38434034

[43] Barik P, Shibu MA, Hsieh DJ, Day CH, Chen RJ, Kuo WW, Huang CY, et al. Cardioprotective effects of transplanted adipose-derived stem cells under Ang II stress with Danggui administration augments cardiac function through upregulation of insulin-like growth factor 1 receptor in late-stage hypertension rats. Environ Toxicol. 2021;36(7):1466–75. 33881220 10.1002/tox.23145

[44] Ball SG, Shuttleworth CA, Kielty CM. Platelet-derived growth factor receptors regulate mesenchymal stem cell fate: implications for neovascularization. Expert Opin Biol Ther. 2010;10(1):57–71. 20078229 10.1517/14712590903379510

[45] Sun C, Liu J, Li N, Liu M, Luo Z, Li H, Chin K-Y. Traditional Chinese Medicine Shenmayizhi Decoction ameliorates memory and cognitive impairment induced by multiple cerebral infarctions. Evidence-Based Complementary and Alternative Medicine. 2021;2021:1–12.

[46] Yin X-X, Chen Z-Q, Liu Z-J, Ma Q-J, Dang G-t. Icariine stimulates proliferation and differentiation of human osteoblasts by increasing production of bone morphogenetic protein 2. Chin Med J (Engl). 2007;120(3):204–10. 17355822

[47] Carreira ACO, Zambuzzi WF, Rossi MC, Filho RA, Sogayar MC. JM Granjeiro. Bone morphogenetic proteins. Bone Morphogenic Protein. Vitamins & Hormones2015. pp. 293–322. 10.1016/bs.vh.2015.06.002 26279381

[48] Ruisi Liu Y, Gong C, Xia Y, Cao C, Zhao M, Zhou. Itaconate: a promising precursor for treatment of neuroinflammation associated depression. Biomed Pharmacother. 2023;167:1–10. 10.1016/j.biopha.2023.115521 37717531

[49] Kevin Perez S, Ciotlos J, Mcgirr C, Limbad R, Doi JP, Nederveen S, et al. Melov: single nuclei profiling identifies cell specific markers of skeletal muscle aging, frailty, and senescence. Aging. 2022;14(23):9393–422. 36516485 10.18632/aging.204435 PMC9792217

[50] Hasegawa MK-HS, Hasebe Y, Inoue Y, Okuno R, Arima M, et al. Akamatsu: increase in inhibin beta A/Activin-A expression in the human epidermis and the suppression of epidermal stem/progenitor cell proliferation with aging. J Dermatol Sci. 2022;106(3):150–8. 35610160 10.1016/j.jdermsci.2022.05.001

[51] João A, Amorim G, Coppotelli AP, Rolo CM, Palmeira JM, Ross. DA sinclair: mitochondrial and metabolic dysfunction in ageing and age-related diseases. Nat Reviews Endocrinol. 2022;18(4):243–58. 10.1038/s41574-021-00626-7 PMC9059418 35145250

[52] Toby Chin XE, Lee O, Dreesen PY, Ng Y, Lee. The role of cellular senescence in skin aging and age-related skin pathologies. Front Physiol. 2023;14:1–15. 10.3389/fphys.2023.1297637 PMC10703490 38074322

[53] Liu B, Qu J, Zhang W, Izpisua Belmonte JC, Liu G-H. A stem cell aging framework, from mechanisms to interventions. Cell Rep. 2022;41(3):1–16. 10.1016/j.celrep.2022.111451 36261013

[54] Ding X, Ma X, Meng P, Yue J, Li L, Xu L. Potential effects of traditional Chinese medicine in anti-aging and aging-related diseases: current evidence and perspectives. Clin Interv Aging. 2024;19:681–93. 38706635 10.2147/CIA.S447514 PMC11070163

[55] Jue Wang Y, Ying Z, Chen K, Shao W, Zhang S, Lin. Guilu erxian glue (龟鹿二仙胶) inhibits chemotherapy-induced bone marrow hematopoietic stem cell senescence in mice may via p16INK4a-Rb signaling pathway. Chin J Integr Med. 2020;26(11):819–24. 32915425 10.1007/s11655-020-3098-3

[56] Liang B, Chen X, Li M, Zhang L, Yang X, Shi L, Yang L, et al. Liuwei Dihuang pills attenuate ovariectomy-induced bone loss by alleviating bone marrow mesenchymal stem cell (BMSC) senescence via the Yes-associated protein (YAP)-autophagy axis. Pharm Biol. 2023;62(1):42–52. 38112463 10.1080/13880209.2023.2291675 PMC11734888

[57] Huang H, Qian Y, Feng Y, Wang Y, Qian P, Xu F, Wang Q. Erxian Decoction-induced serum exosomes slowed bone marrow mesenchymal stem cell senescence through mitophagy. J Gene Med. 2023;26(1):1–14. 10.1002/jgm.3617 37935422

[58] Ke H, Zhang X, Liang S, Zhou C, Hu Y, Huang Q, Wu J. Study on the anti-skin aging effect and mechanism of Sijunzi Tang based on network pharmacology and experimental validation. J Ethnopharmacol. 2024;333:1–14. 10.1016/j.jep.2024.118421 38880400

[59] Yinan Liu Y, Liu X, Wang C, Xiu, Yanhong H, Wang J, Yang J, et al. Ginseng-Sanqi-Chuanxiong (GSC) extracts attenuate d-galactose-induced vascular aging in mice via inhibition of endothelial progenitor cells senescence. Heliyon. 2024;10(4):1–13. 10.1016/j.heliyon.2024.e25253 PMC10884806 38404901

[60] Ho T-J, Goswami D, Kuo W-W, Kuo C-H, Yen SC, Lin P-Y, et al. C-Y huang: Artemisia argyi exhibits anti-aging effects through decreasing the senescence in aging stem cells. Aging. 2022;14(15):6187–201. 35951373 10.18632/aging.204210 PMC9417221

[61] Lee P-Y, Tsai BC-K, Sitorus MA, Lin P-Y, Lin S-Z, Shih C-Y, et al. C-Y huang: Ohwia caudata aqueous extract attenuates doxorubicin-induced mitochondrial dysfunction in wharton’s jelly‐derived mesenchymal stem cells. Environ Toxicol. 2023;38(10):2450–61. 37461261 10.1002/tox.23880

[62] Ying Liu X, Wang C, Jin J, Qiao C, Wang L, Jiang M, et al. Liu: total ginsenosides extend healthspan of aging drosophila by suppressing imbalances in intestinal stem cells and microbiota. Phytomedicine. 2024;129:1–16. 10.1016/j.phymed.2024.155650 38669971

[63] Weng Z, Wang Y, Ouchi T, Liu H, Qiao X, Wu C, et al. Li: mesenchymal stem/stromal cell senescence: hallmarks, mechanisms, and combating strategies. Stem Cells Translational Med. 2022;11(4):356–71. 10.1093/stcltm/szac004 PMC9052415 35485439

[64] Zhao J, Lan X, Liu Y, Liu Y, Xian Y, Lin Z, Xu F. Anti-aging role of Chinese Herbel medicine: an overview of scientific evidence from 2008 to 2018. Annals Palliat Med. 2020;9(3):1230–48. 10.21037/apm.2020.04.09 32389009

[65] Yang H, Yang Y, Kiskin FN, Shen M, Zhang JZ. Recent advances in regulating the proliferation or maturation of human-induced pluripotent stem cell-derived cardiomyocytes. Stem Cell Res Ther. 2023;14(1):1–17. 37649113 10.1186/s13287-023-03470-w PMC10469435

[66] Cuesta-Gomez N, Verhoeff K, Jasra IT, Pawlick R, Dadheech N, Shapiro AMJ. Characterization of stem-cell-derived islets during differentiation and after implantation. Cell Rep. 2022;40(8):1–13. 10.1016/j.celrep.2022.111238 36001981

[67] Ningning Yang G, Liang J, Lin S, Zhang Q, Lin X, Ji S, Jin, et al. Ginsenoside Rd therapy improves histological and functional recovery in a rat model of inflammatory bowel disease. Phytother Res. 2020;34(11):3019–28. 32468636 10.1002/ptr.6734

[68] Tao Q, Zhang J, Liang Q, Song S, Wang S, Yao X, Wang L, et al. Puerarin alleviates sleep disorders in aged mice related to repairing intestinal mucosal barrier. Nat Prod Bioprospecting. 2023;13(1):1–13. 10.1007/s13659-023-00390-3 PMC10497485 37698689

[69] Xiaoyu Y, Hui X, Xiaolong L, Lili X, Zhuo Y, Xinyu Q. The mechanism of Danzikang knee granule in regulating the chondrogenic differentiation of mesenchymal stem cells based on TGF-β signaling pathway in cartilage repair in knee osteoarthritis. Cell Mol Biol. 2022;67(5):164–73. 35818257 10.14715/cmb/2021.67.5.23

[70] Sheng Zheng G, Hu J, Li J, Zheng Y, Li. Icariin accelerates bone regeneration by inducing osteogenesis-angiogenesis coupling in rats with type 1 diabetes mellitus. World J Diabetes. 2024;15(4):769–82. 38680705 10.4239/wjd.v15.i4.769 PMC11045423

[71] Wei Qin S, Chen S, Yang Q, Xu C, Xu J, Cai. The effect of traditional Chinese medicine on neural stem cell proliferation and differentiation. Aging Disease. 2017;8(6):792–811. 29344417 10.14336/AD.2017.0428 PMC5758352

[72] Zhang M, Chai Y, Liu T, Xu N, Yang C. Synergistic effects of Buyang Huanwu Decoction and embryonic neural stem cell transplantation on the recovery of neurological function in a rat model of spinal cord injury. Experimental Therapeutic Med. 2015;9(4):1141–8. 10.3892/etm.2015.2248 PMC4353797 25780400

[73] Ziqiao Y, Yin B, Yuguowang Z, Ni J, Feng Q, Yang Y, Dou, et al. Therapeutic mechanism of Liangxue-Guyuan-Yishen Decoction on intestinal stem cells and tight junction proteins in gastrointestinal acute radiation syndrome rats. J Radiat Res. 2023;64(6):880–92. 37697698 10.1093/jrr/rrad065 PMC10665307

[74] Ma H, Yue GGar–Lee, Lee JK-M, Gao S, Yuen K-K, Cheng W, et al. CBS lau: scutellarin, a flavonoid compound from scutellaria barbata, suppresses growth of breast cancer stem cells in vitro and in tumor-bearing mice. Phytomedicine. 2024;128:1–14. 10.1016/j.phymed.2024.155418 38518647

[75] Cucco C, Zhang Z, Botero TM, Chiego DJ, Rogerio M, Castilho. JE nöra: SCF/C-Kit signaling induces self-renewal of dental pulp stem cells. J Endod. 2020;46(9):S56–62. 32950196 10.1016/j.joen.2020.06.035 PMC7508352

[76] Liangliang Niu Y, Fang X, Yao Y, Zhang J, Wu DF, Chen X, Sun. TNFα activates MAPK and Jak-Stat pathways to promote mouse Müller cell proliferation. Exp Eye Res. 2021;202:1–11. 10.1016/j.exer.2020.108353 33171193

[77] Jason SL, Yu W, Cui. Proliferation, survival and metabolism: the role of PI3K/AKT/mTOR signalling in pluripotency and cell fate determination. Development. 2016;143(17):3050–60. 27578176 10.1242/dev.137075

[78] Yu S, Liu W, Liu T, Feng X, Yang N, Zhou H. Signaling pathway of MAPK/ERK in cell proliferation, differentiation, migration, senescence and apoptosis. J Recept Signal Transduct Res. 2015;35(6):600–4. 26096166 10.3109/10799893.2015.1030412

[79] Eudald Pascual-Carreras, Miquel Sureda-Gómez, Ramon Barrull-Mascaró, Natàlia Jordà, Maria Gelabert, Pablo Coronel-Córdoba, et al T Adell: WNT-FRIZZLED-LRP5/6 signaling mediates posterior fate and proliferation during planarian regeneration. Genes. 2021;12(1):1–14. 10.3390/genes12010101 PMC7830089 33467529

[80] Jacob P, Mahoney ES, Bruguera M, Vasishtha LB, Killingsworth S, Kyaw WI, Weis. PI(4,5)P_2_-stimulated positive feedback drives the recruitment of dishevelled to frizzled in Wnt–β-catenin signaling. Sci Signal. 2022;15(748):1–30. 10.1126/scisignal.abo2820 PMC9528458 35998232

[81] Wang P, Xu T, Yan Z, Zheng X, Zhu F. Jian-Pi-Yi-Qi-Fang ameliorates chronic atrophic gastritis in rats through promoting the proliferation and differentiation of gastric stem cells. Annals Translational Med. 2022;10(17):1–18. 10.21037/atm-22-3749 PMC9511200 36172111

[82] Tan Y-l, Ju S-h, Wang Q, Zhong R, Gao J-h, Wang M-j, et al. M-z xu: Shuanglongjiegu pill promoted bone marrow mesenchymal stem cell osteogenic differentiation by regulating the miR-217/RUNX2 axis to activate Wnt/β-catenin pathway. J Orthop Surg Res. 2024;19(1):1–11. 39350234 10.1186/s13018-024-05085-0 PMC11443779

[83] Qian H, Ye Z, Hu Y, Chen L, Li L, Qin K, et al. X zuo: Dahuang-Gancao Decoction ameliorates testosterone-induced androgenetic alopecia in mice. J Ethnopharmacol. 2025;341:1–15. 10.1016/j.jep.2025.119347 39800247

[84] Luo Z, Li Z, Liang Z, Wang L, He G, Wang D, Li B, et al. Berberine increases stromal production of Wnt molecules and activates Lgr5 + stem cells to promote epithelial restitution in experimental colitis. BMC Biol. 2022;20(1):1–19. 36528592 10.1186/s12915-022-01492-z PMC9759859

[85] Xu W, Du X, Li J, Zhang Z, Ma X, Luo D, et al. Q sun: SiNiSan alleviates liver injury by promoting hepatic stem cell differentiation via Wnt/β-catenin signaling pathway. Phytomedicine. 2022;99:1–13. 10.1016/j.phymed.2022.153969 35183930

[86] Ziling Wang R, Jiang L, Wang X, Chen Y, Xiang L, Chen Y, Wang et al. Ginsenoside Rg1 improves differentiation by inhibiting senescence of human bone marrow mesenchymal stem cell via GSK-3β and β-Catenin. Stem Cells International. 2020;2020:1–16. 10.1155/2020/2365814 PMC7271209 32565825

[87] Lulu Guo R, Du YZ, Li H, Li G, Wu S. Ginseng promotes the function of intestinal stem cells through the Wnt/β-catenin signaling pathway in D-galactose-induced aging mice. Exp Gerontol. 2024;185:1–11. 10.1016/j.exger.2023.112351 38135257

[88] Zhang S, Lin K, Law CY, Liu B, Fu X, Tse W, et al. KKL yung: oleanolic acid enhances neural stem cell migration, proliferation, and differentiation in vitro by inhibiting GSK3β activity. Cell Death Discovery. 2018;4(1):1–9. 10.1038/s41420-018-0111-0 PMC6189131 30345079

[89] Cui Wang J, Chen H, Xiao L, Kong Y, Zhao Y, Tian D, Shang, et al. Jujuboside a promotes proliferation and neuronal differentiation of APPswe-overexpressing neural stem cells by activating Wnt/β-catenin signaling pathway. Neurosci Lett. 2022;772:1–10. 10.1016/j.neulet.2022.136473 35077846

[90] Honghe Xiao M, Zhang J, Xu Y, Deng N, Li P, Gao J, Yang, et al. Icarisid II promotes proliferation and neuronal differentiation of neural stem cells via activating Wnt/β-catenin signaling pathway. Phytother Res. 2021;35(5):2773–84. 33455039 10.1002/ptr.7022

[91] Hayat R, Manzoor M, Hussain A. Wnt signaling pathway: a comprehensive review. Cell Biol Int. 2022;46(6):863–77. 35297539 10.1002/cbin.11797

[92] Fu Y, Kong Y, Li J, Wang Y, Li M, Wang Y, et al. Z chang: mesenchymal stem cells combined with traditional Chinese medicine (qi-fang-bi-min-tang) alleviates rodent allergic rhinitis. J Cell Biochem. 2020;121(2):1541–51. 31535402 10.1002/jcb.29389

[93] Zhang S, Zhan J, Li M, Wang J, Chen H, Wang Y, Wu C, et al. Therapeutic potential of traditional Chinese medicine against osteoarthritis: targeting the Wnt signaling pathway. Am J Chin Med. 2024;52(7):2021–52. 39562354 10.1142/S0192415X24500782

[94] Wu S, Ge Y, Lin K, Liu Q, Zhou H, Hu Q, et al. Z ju: telomerase RNA TERC and the PI3K-AKT pathway form a positive feedback loop to regulate cell proliferation independent of telomerase activity. Nucleic Acids Res. 2022;50(7):3764–76. 35323972 10.1093/nar/gkac179 PMC9023280

[95] Liu Y, Yu H, Nimer SD. PI3K-Akt pathway regulates polycomb group protein and stem cell maintenance. Cell Cycle. 2014;12(2):199–200. 10.4161/cc.23379 PMC3575444 23287474

[96] Jingjing Jiang N, Fu X, Sang S, Zheng Q, Zhao. The n-butanol extract of Gualou Xiebai Banxia Decoction inhibits ischemia-hypoxia-induced apoptosis of bone marrow mesenchymal stem cells through the PI3K/AKT signaling pathway. Pharmacol Clin Chin Materia Med. 2021;37(4):2–7.

[97] Tian Q, Zhang L, Long Y. Study on the effect of astragalus on proliferation of bone marrow mesenchymal stem cells in anoxic microenvironment based on PI3K-AKT signal pathway. Acta Vet Et Zootechnica Sinica. 2024;55(1):346–54.

[98] Liu Y, Yongge G, Yang S. Guishen pill water extract intervenes with the proliferation of bone marrow mesenchymal stem cells and expression levels of PI3K and AKT in Sprague-Dawley rats. Chin J Tissue Eng Res. 2020;24(13):1983–8.

[99] Qiaohui Du R, Deng W, Li D, Zhang B, Tsoi J, Shen. Baoyuan capsule promotes neurogenesis and neurological functional recovery through improving mitochondrial function and modulating PI3K/Akt signaling pathway. Phytomedicine. 2021;93:1–12. 10.1016/j.phymed.2021.153795 34735905

[100] Cai B, Jin B, Sun D, Deng W. Yangjing capsule extract promotes proliferation of spermatogonia stem cells via PI3K-Akt-Cyclin D1 pathway. Chin J Androl. 2020;34(6):24–9.

[101] Ma Y, Lang X, Yang Q, Han Y, Kang X, Long R, Liu J, et al. Paeoniflorin promotes intestinal stem cell-mediated epithelial regeneration and repair via PI3K-AKT-mTOR signalling in ulcerative colitis. Int Immunopharmacol. 2023;119:110247. 37159966 10.1016/j.intimp.2023.110247

[102] Liu Lan W, Song Z, Cheng M, Yongping. Berberine regulates the proliferation and osteogenic differentiation of human periodontal ligament stem cells through PI3K/AKT signaling pathway. Chin J Cell Biology. 2024;46(3):494–501.

[103] Ziling Wang L, Wang R, Jiang C, Li X, Chen H, Xiao Y, Wang, et al. Ginsenoside Rg1 prevents bone marrow mesenchymal stem cell senescence via NRF2 and PI3K/Akt signaling. Free Radic Biol Med. 2021;174:182–94. 34364981 10.1016/j.freeradbiomed.2021.08.007

[104] Cai H, Han X-j, Luo Z-r, Wang Q-l, Lu P-p, Mou F-f, et al. H-d guo: pretreatment with notoginsenoside R1 enhances the efficacy of neonatal rat mesenchymal stem cell transplantation in model of myocardial infarction through regulating PI3K/Akt/FoxO1 signaling pathways. Stem Cell Res Ther. 2024;15(1):1–17. 39533348 10.1186/s13287-024-04039-x PMC11558819

[105] Zou Y, Li Z, Lin Y, Zheng Y, Liu Z, Li Y, Zhu L, et al. Shanyao regulates the PI3K/AKT/P21 pathway to promote oogonial stem cell proliferation and stemness restoration to alleviate premature ovarian insufficiency. J Ethnopharmacol. 2025;340:119168. 39615771 10.1016/j.jep.2024.119168

[106] Xu J, Liu X, Jiang Y, Chu L, Hao H, Liua Z, et al. Z liu: MAPK/ERK signalling mediates VEGF-induced bone marrow stem cell differentiation into endothelial cell. J Cell Mol Med. 2008;12(6a):2395–406. 18266967 10.1111/j.1582-4934.2008.00266.x PMC4514117

[107] Jiang J, Hai J, Liu W, Luo Y, Chen K, Xin Y, et al. Luo: gallic acid induces neural stem cell differentiation into neurons and proliferation through the MAPK/ERK pathway. J Agric Food Chem. 2021;69(42):12456–64. 34647728 10.1021/acs.jafc.1c04011

[108] Chen X, Wu H, Chen H, Wang Q, Xie X, Jiangang S. Astragaloside VI promotes neural stem cell proliferation and enhances neurological function recovery in transient cerebral ischemic injury via activating EGFR/MAPK signaling cascades. Mol Neurobiol. 2018;56(4):3053–67. 30088176 10.1007/s12035-018-1294-3

[109] Yongtao Niu L, Xie R, Deng X, Zhang. In the presence of TGF-β1, Asperosaponin VI promotes human mesenchymal stem cell differentiation into nucleus pulposus like-cells. BMC Complement Med Ther. 2021;21(1):1–11. 33446173 10.1186/s12906-020-03169-y PMC7807821

[110] Li K, Ma X, Li Z, Liu Y, Shen G, Luo Z, Li B, et al. A natural peptide from A traditional Chinese medicine has the potential to treat chronic atrophic gastritis by activating gastric stem cells. Adv Sci. 2024;11(20):1–15. 10.1002/advs.202304326 PMC11132046 38544338

[111] Tu S, Huang Y, Tian H, Xu L, Wang X, Huang L, et al. D liu: Berberine enhances the function of intestinal stem cells in healthy and radiation-injured mice. Int Immunopharmacol. 2024;136:1–12. 10.1016/j.intimp.2024.112278 38815353

[112] Bai Y, Zhong Z, Song C. Effects of naringin on MAPK signaling pathway in promoting osteogenic differentiation of rat bone marrow mesenchymal stem cells. Chin Remedies Clin. 2024;24(2):107–10.

[113] Shen L, Han F, Pan L, Du L, Sun P, Zhang K, Zhu J, et al. Construction of tissue engineered cornea with skin-derived corneal endothelial-like cell and mechanism research for the cell differentiation. Front Med. 2024;11:1–16. 10.3389/fmed.2024.1448248 PMC11402686 39286645

[114] Zhou L, Li M, Chai Z, Zhang J, Cao K, Deng L, et al. Han: anticancer effects and mechanisms of astragaloside–IV (Review). Oncol Rep. 2022;49(1):1–16. 36367181 10.3892/or.2022.8442 PMC9685271

[115] Tao Z, Tan S, Chen W, Chen X. Stem cell homing: a potential therapeutic strategy unproven for treatment of myocardial injury. J Cardiovasc Transl Res. 2018;11(5):403–11. 30324254 10.1007/s12265-018-9823-z

[116] Liesveld JL, Sharma N, Aljitawi OS. Stem cell homing: from physiology to therapeutics. Stem Cells. 2020;38(10):1241–53. 32526037 10.1002/stem.3242

[117] Tsvee Lapidot I, Petit. Current understanding of stem cell mobilization: the roles of chemokines, proteolytic enzymes, adhesion molecules, cytokines, and stromal cells. Exp Hematol. 2002;30(9):973–81. 12225788 10.1016/s0301-472x(02)00883-4

[118] Zhang H, Bai H, Yi Z, He X, Mo S. Effect of stem cell factor and granulocyte-macrophagecolony-stimulating factor-induced bone marrow stem cellMobilization on recovery from acute tubular necrosis in rats. Ren Fail. 2012;34(3):350–7. 22260331 10.3109/0886022X.2011.647340

[119] Herbert KE, Morgan S, Prince HM, Westerman DA, Wolf MM, Carney DA, Seymour JF, et al. Stem cell factor and high-dose twice daily filgrastim is an effective strategy for peripheral blood stem cell mobilization in patients with indolent lymphoproliferative disorders previously treated with fludarabine: results of a phase II study with an historical comparator. Leukemia. 2009;23(2):305–12. 18987661 10.1038/leu.2008.302

[120] Zhang J-S, Zhang B-X, Du M-M, Wang X-Y, Li W. Chinese preparation Xuesaitong promotes the mobilization of bone marrow mesenchymal stem cells in rats with cerebral infarction. Neural Regeneration Res. 2016;11(2):292–7. 10.4103/1673-5374.177738 PMC4810994 27073383

[121] Wanting Meng Z, Xiao H, Li Y, Wang Y, Zhao Y, Zhu H, Guo. Modified Taohong Siwu Decoction improves cardiac function after myocardial ischaemia and reperfusion in rats by promoting endogenous stem cell mobilization and regulating metabolites. Pharm Biol. 2022;60(1):1721–31. 36086864 10.1080/13880209.2022.2116054 PMC9467615

[122] Tang J-M, Wang J-N, Zhang L, Zheng F, Yang J-Y, Kong X, et al. S-Y chen: VEGF/SDF-1 promotes cardiac stem cell mobilization and myocardial repair in the infarcted heart. Cardiovascular Res. 2011;91(3):402–11. 10.1093/cvr/cvr053 PMC3139446 21345805

[123] Shi W-L, Zhao J, Yuan R, Lu Y, Xin Q-Q, Liu Y et al. K-J Chen: combination of Ligusticum ChuanxiongandRadix PaeoniaPromotes angiogenesis in ischemic myocardium through notch signalling and mobilization of stem cells. Evidence-Based Complementary and Alternative Medicine. 2019;2019:1–12.

[124] Huang Y, Hu R, Liu Z, Geng Y, Li F, Song Y, Song K, et al. Bushen Huoxue recipe ameliorates ovarian function via promoting BMSCs proliferation and homing to ovaries in POI mice. Phytomedicine. 2024;129(155630):1–17. 10.1016/j.phymed.2024.155630 38678952

[125] Wang F, Dai H, Zhou Z, Shan Y, Yu M, Sun J, Sheng M, et al. Astragalus polysaccharides augment BMSC homing via SDF-1/CXCR4 modulation: a novel approach to counteract peritoneal mesenchymal transformation and fibrosis. BMC Complement Med Ther. 2024;24(1):1–15. 38789949 10.1186/s12906-024-04483-5 PMC11127382

[126] Gong J, Meng H-B, Hua JIE, Song Z-S, He Z-G, Zhou BO, Qian M-P. The SDF-1/CXCR4 axis regulates migration of transplanted bone marrow mesenchymal stem cells towards the pancreas in rats with acute pancreatitis. Mol Med Rep. 2014;9(5):1575–82. 24626964 10.3892/mmr.2014.2053 PMC4020475

[127] Xiang X, Liu H, Wang L, Zhu B, Ma L, Du F, et al. L qiu: ultrasound combined with SDF-1α chemotactic microbubbles promotes stem cell homing in an osteoarthritis model. J Cell Mol Med. 2020;24(18):10816–29. 33140920 10.1111/jcmm.15706 PMC7521263

[128] Chen J, Wei J, Huang Y, Ma Y, Ni J, Li M, et al. Fan: Danhong injection enhances the therapeutic efficacy of mesenchymal stem cells in myocardial infarction by promoting angiogenesis. Front Physiol. 2018;9:1–12. 30093864 10.3389/fphys.2018.00991 PMC6070728

[129] Han X-j, Li H, Liu C-b, Luo Z-r, Wang Q-l, Mou F-f. H-d guo: Guanxin Danshen formulation improved the effect of mesenchymal stem cells transplantation for the treatment of myocardial infarction probably via enhancing the engraftment. Life Sci. 2019;233:1–10. 10.1016/j.lfs.2019.116740 31398416

[130] Wu KH, Mo XM, Han ZC, Zhou B. Stem cell engraftment and survival in the ischemic heart. Ann Thorac Surg. 2011;92(5):1917–25. 21955573 10.1016/j.athoracsur.2011.07.012

[131] Wang X, Ma S, Yang B, Huang T, Meng N, Xu L, Wang J, et al. Resveratrol promotes hUC-MSCs engraftment and neural repair in a mouse model of alzheimer’s disease. Behav Brain Res. 2018;339:297–304. 29102593 10.1016/j.bbr.2017.10.032 PMC5729114

[132] Wu X-l, Zhuang H-f, Zhao Y-n, Yu X-l, Dai T-y. R-l gao: Chinese medicine treatment on graft-versus-host disease after allogeneic hematopoietic stem cell transplantation. Chin J Integr Med. 2020;26(5):324–9. 32350801 10.1007/s11655-020-3252-y

[133] Ma Z-Y, Zhong Z-G, Qiu M-Y, Zhong Y-H, Zhang W-X. TGF-β1 activates the canonical NF-κB signaling to promote cell survival and proliferation in dystrophic muscle fibroblasts in vitro. Biochem Biophys Res Commun. 2016;471(4):576–81. 26874278 10.1016/j.bbrc.2016.02.029

[134] Pulkkinen HH, Kiema M, Lappalainen JP, Toropainen A, Beter M, Tirronen A et al. JP Laakkonen: BMP6/TAZ-Hippo signaling modulates angiogenesis and endothelial cell response to VEGF. Angiogenesis. 2020;24(1):129–144. 10.1007/s10456-020-09748-4 PMC7921060 33021694

[135] Song X, Qu H, Wang Y, Yang T, Rong J, Zhou H. What can we do to optimize stem and progenitor cell therapy for heart failure? Discov Med. 2018;25(137):113–30. 29641973

[136] Li Z, Zhang M, Zheng J, Tian Y, Zhang H, Tan Y, et al. Huang: human umbilical cord mesenchymal stem cell-derived exosomes improve ovarian function and proliferation of premature ovarian insufficiency by regulating the Hippo signaling pathway. Front Endocrinol. 2021;12:1–17. 10.3389/fendo.2021.711902 PMC8397419 34456868

[137] Wang J, Wu H, Peng Y, Zhao Y, Qin Y, Zhang Y, Xiao Z. Hypoxia adipose stem cell-derived exosomes promote high-quality healing of diabetic wound involves activation of PI3K/Akt pathways. J Nanobiotechnol. 2021;19(1):1–13. 10.1186/s12951-021-00942-0 PMC8261989 34233694

[138] Fei Tan X, Li Z, Wang J, Li K, Shahzad J, Zheng. Clinical applications of stem cell-derived exosomes. Signal Transduct Target Therapy. 2024;9(1):1–31. 10.1038/s41392-023-01704-0 PMC10784577 38212307

[139] Hade MD, Suire CN, Suo Z. Mesenchymal stem cell-derived exosomes: applications in regenerative medicine. Cells. 2021;10(8):1–48. 10.3390/cells10081959 PMC8393426 34440728

[140] Liu W-z, Ma Z-j, Li J-r, Kang X-w. Mesenchymal stem cell-derived exosomes: therapeutic opportunities and challenges for spinal cord injury. Stem Cell Res Ther. 2021;12(1):1–15. 33536064 10.1186/s13287-021-02153-8 PMC7860030

[141] Guo G, Tan Z, Liu Y, Shi F, She J. The therapeutic potential of stem cell-derived exosomes in the ulcerative colitis and colorectal cancer. Stem Cell Res Ther. 2022;13(1):1–18. 35365226 10.1186/s13287-022-02811-5 PMC8973885

[142] He L, He T, Xing J, Zhou Q, Fan L, Liu C, et al. Rong: bone marrow mesenchymal stem cell-derived exosomes protect cartilage damage and relieve knee osteoarthritis pain in a rat model of osteoarthritis. Stem Cell Res Ther. 2020;11(1):1–15. 32650828 10.1186/s13287-020-01781-w PMC7350730

[143] Chen DY, Li BZ, Xu WB, Zhang YM, Li BW, Cheng YX, et al. Shu: the first identification of three AdIRAK2 genes from an evolutionarily important amphibian Andrias Davidianus and their involvement in NF-κB activation and inflammatory responses. Dev Comp Immunol. 2023;139:104585. 36368593 10.1016/j.dci.2022.104585

[144] Yuyan Xiong R, Tang J, Xu W, Jiang Z, Gong L, Zhang Y, Yang, et al. Tongxinluo-pretreated mesenchymal stem cells facilitate cardiac repair via exosomal transfer of miR-146a-5p targeting IRAK1/NF-κB p65 pathway. Stem Cell Res Ther. 2022;13(1):1–18. 35799283 10.1186/s13287-022-02969-y PMC9264662

[145] Li S, Cao W, Guo R, Liu Z, Zhang J. G fan: Tanshinone IIA enhances the therapeutic efficacy of mesenchymal stem cells derived exosomes in myocardial ischemia/reperfusion injury via upregulating miR-223-5p. J Controlled Release. 2021;358:13–26. 10.1016/j.jconrel.2023.04.014 37086952

[146] Li R, Duan W, Feng T, Gu C, Zhang Q, Chen JL, et al. Lycium barbarum polysaccharide inhibits ischemia-induced autophagy by promoting the biogenesis of neural stem cells-derived extracellular vesicles to enhance the delivery of miR-133a-3p. Chin Med. 2023;18(1):1–16. 37691119 10.1186/s13020-023-00831-8 PMC10494430

[147] Shengxi Meng H, Chen C, Deng Z, Meng. Catalpol mitigates alzheimer’s disease progression by promoting the expression of neural stem cell exosomes released miR-138-5p. Neurotox Res. 2023;41(1):41–56. 36595161 10.1007/s12640-022-00626-z PMC9944361

[148] Yu J, Wu X, Zhang W, Chu F, Zhang Q, Gao M, Wu Y, et al. Effect of psoralen on the regulation of osteogenic differentiation induced by periodontal stem cell-derived exosomes. Hum Cell. 2023;36(4):1389–402. 37269415 10.1007/s13577-023-00918-2 PMC10284944

[149] Zhao Y-H, Guan Y, Wu W-k. Potential advantages of a combination of Chinese medicine and bone marrow mesenchymal stem cell transplantation for removing blood stasis and stimulating neogenesis during ischemic stroke treatment. J Tradit Chin Med. 2012;32(2):289–92. 22876459 10.1016/s0254-6272(13)60027-8

[150] Liao Y, Luo Z, Liu Y, Xue W, He S, Chen X, Guo H, et al. Total flavonoids of litchi seed attenuate stem cell-like properties in breast cancer by regulating Notch3 signaling pathway. J Ethnopharmacol. 2023;305:1–12. 10.1016/j.jep.2023.116133 36603788

[151] Zhao P, Li J, Yang L, Li Y, Tian Y, Li S. Integration of transcriptomics, proteomics, metabolomics and systems pharmacology data to reveal the therapeutic mechanism underlying Chinese herbal bufei Yishen formula for the treatment of chronic obstructive pulmonary disease. Mol Med Rep. 2018;17(4):5247–57. 10.3892/mmr.2018.8480 PMC5865990 29393428

[152] Lin L, Tang R, Liu Y, Li Z, Li H, Yang H. Research on the anti-aging mechanisms of Panax ginseng extract in mice: a gut microbiome and metabolomics approach. Front Pharmacol. 2024;15:1–18. 10.3389/fphar.2024.1415844 PMC11222675 38966558

[153] Zhang Q, Han W, Wu R, Deng S, Meng J, Yang Y, Li H, et al. Spermidine-eIF5A axis is essential for muscle stem cell activation via translational control. Cell Discovery. 2024;10(1):1–19. 39251577 10.1038/s41421-024-00712-w PMC11383958

[154] Chang X, Wu D, Gao X, Lin J, Tan Y, Wang J, et al. Zhou: BuyangHuanwu Decoction alleviates endothelial cell apoptosis and coronary microvascular dysfunction via regulation of the MAPKK4/p38 signaling axis. Int J Med Sci. 2024;21(13):2464–79. 39439466 10.7150/ijms.98183 PMC11492876

[155] Wang Z, Han T, Zhu H, Tang J, Guo Y, Jin Y, et al. Wang: potential osteoinductive effects of hydroxyapatite nanoparticles on mesenchymal stem cells by endothelial cell interaction. Nanoscale Res Lett. 2021;16(1):1–16. 33900483 10.1186/s11671-021-03522-1 PMC8076414

